# Deciphering genetic and nongenetic factors underlying tumour dormancy: insights from multiomics analysis of two syngeneic MRD models of melanoma and leukemia

**DOI:** 10.1186/s40659-024-00540-y

**Published:** 2024-09-03

**Authors:** Marie-Océane Laguillaumie, Sofia Titah, Aurélie Guillemette, Bernadette Neve, Frederic Leprêtre, Pascaline Ségard, Faruk Azam Shaik, Dominique Collard, Jean-Claude Gerbedoen, Léa Fléchon, Lama Hasan Bou Issa, Audrey Vincent, Martin Figeac, Shéhérazade Sebda, Céline Villenet, Jérôme Kluza, William Laine, Isabelle Fournier, Jean-Pascal Gimeno, Maxence Wisztorski, Salomon Manier, Mehmet Cagatay Tarhan, Bruno Quesnel, Thierry Idziorek, Yasmine Touil

**Affiliations:** 1 CNRS, Inserm, CHU Lille, Institut Pasteur de Lille, UMR9020-U1277-CANTHER-Cancer Heterogeneity Plasticity and Resistance to Therapies, Univ. Lille, 59000 Lille, France; 2 Inserm, U1003-PHYCEL-Physiologie Cellulaire, Univ. Lille, 59000 Lille, France; 3 CNRS, Inserm, CHU Lille, Institut Pasteur de Lille, US 41-UAR 2014-PLBS, Univ. Lille, 59000 Lille, France; 4 LIMMS/CNRS-IIS IRL2820, The University of Tokyo, Tokyo, Japan; 5 CNRS, IIS, COL, Univ. Lille SMMiL-E Project, Lille, France; 6 Department of Health and Environment, Junia HEI-ISEN-ISA, Lille, France; 7 Inserm, CHU Lille, U1192, Laboratoire Protéomique, Réponse Inflammatoire Et Spectrométrie de Masse (PRISM), Univ. Lille, 59000 Lille, France; 8 CNRS, Centrale Lille, Polytechnique Hauts-de-France, Junia, UMR 8520-IEMN, Univ. Lille, Villeneuve d’Ascq, France

**Keywords:** Tumour dormancy, Leukemia, Melanoma, Syngeneic model, Multiomics analysis, ChIP-seq, Whole exome sequencing, Copy number variation

## Abstract

**Background:**

Tumour dormancy, a resistance mechanism employed by cancer cells, is a significant challenge in cancer treatment, contributing to minimal residual disease (MRD) and potential relapse. Despite its clinical importance, the mechanisms underlying tumour dormancy and MRD remain unclear. In this study, we employed two syngeneic murine models of myeloid leukemia and melanoma to investigate the genetic, epigenetic, transcriptomic and protein signatures associated with tumour dormancy. We used a multiomics approach to elucidate the molecular mechanisms driving MRD and identify potential therapeutic targets.

**Results:**

We conducted an in-depth omics analysis encompassing whole-exome sequencing (WES), copy number variation (CNV) analysis, chromatin immunoprecipitation followed by sequencing (ChIP-seq), transcriptome and proteome investigations. WES analysis revealed a modest overlap of gene mutations between melanoma and leukemia dormancy models, with a significant number of mutated genes found exclusively in dormant cells. These exclusive genetic signatures suggest selective pressure during MRD, potentially conferring resistance to the microenvironment or therapies. CNV, histone marks and transcriptomic gene expression signatures combined with Gene Ontology (GO) enrichment analysis highlighted the potential functional roles of the mutated genes, providing insights into the pathways associated with MRD. In addition, we compared “murine MRD genes” profiles to the corresponding human disease through public datasets and highlighted common features according to disease progression. Proteomic analysis combined with multi-omics genetic investigations, revealed a dysregulated proteins signature in dormant cells with minimal genetic mechanism involvement. Pathway enrichment analysis revealed the metabolic, differentiation and cytoskeletal remodeling processes involved in MRD. Finally, we identified 11 common proteins differentially expressed in dormant cells from both pathologies.

**Conclusions:**

Our study underscores the complexity of tumour dormancy, implicating both genetic and nongenetic factors. By comparing genomic, transcriptomic, proteomic, and epigenomic datasets, our study provides a comprehensive understanding of the molecular landscape of minimal residual disease. These results provide a robust foundation for forthcoming investigations and offer potential avenues for the advancement of targeted MRD therapies in leukemia and melanoma patients, emphasizing the importance of considering both genetic and nongenetic factors in treatment strategies.

**Graphical Abstract:**

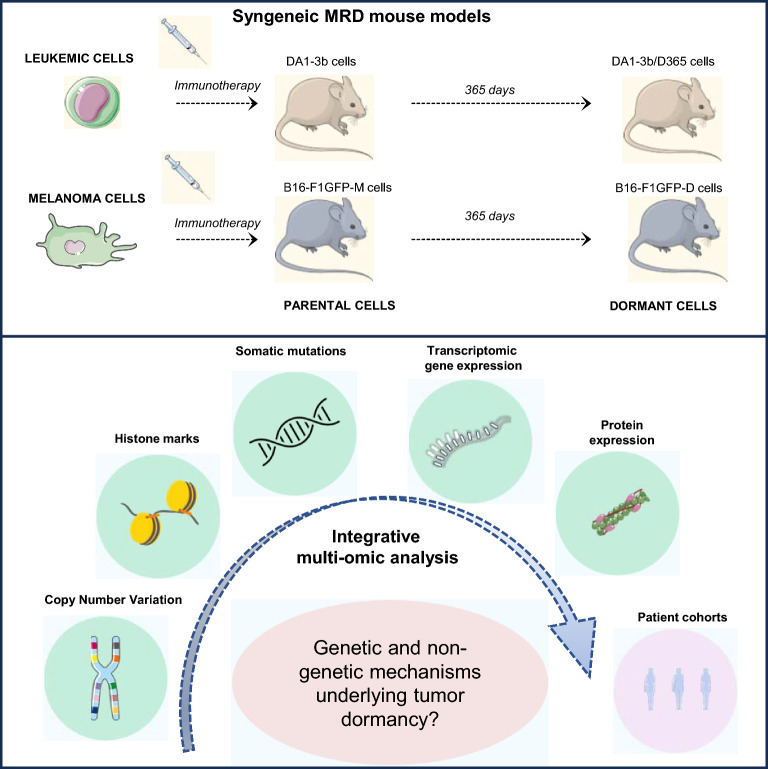

**Supplementary Information:**

The online version contains supplementary material available at 10.1186/s40659-024-00540-y.

## Introduction

Tumour dormancy is one of the resistance mechanisms that tumour cells use to persist within the body for extended periods, spanning months to even years [1–3]. Their occurrence is observed in healthy individuals and cancer patients, and persists even after treatment [4, 5]. Dormant cancer cells are able to escape the immune system and resist therapies; this event is termed minimal residual disease (MRD) [1, 2, 6]. Given that tumour dormancy contributes markedly to relapse, a thorough knowledge of its mechanisms is crucial for developing effective therapeutic strategies aimed at improving patient outcomes.

MRD phenomenon has been described in both solid tumours and hematologic malignancies [1, 6]. Generally, intra- and interpatient tumour heterogeneity arises from mutations, which can impact patient outcomes and be useful prognostic indicators for therapeutic decisions. However, the specific influence of these mutations on tumour dormancy remains unclear. In addition, the limited number of studies describing MRD models did not reveal sufficient knowledge of tumour dormancy mechanisms [7]. Although studies have revealed robust specific signatures of dormant or residual cells, the data are often limited to DNA and/-or RNA sequencing minimizing the complexity between the MRD process and the immune system [8–10].

In this context, our laboratory has developed two syngeneic mouse models of myeloid leukemia and melanoma tumour dormancy. As previously described, these models were established through cellular vaccination with irradiated cells overexpressing interleukin-12 (IL-12) or granulocyte–macrophage colony-stimulating factor (GM-CSF), in leukemia and melanoma respectively [11, 12]. With the DA1-3b leukemic model, we isolated dormant cells at different time point during the MRD process [11]. Similarly, we established B16-F1 murine model of dormant melanoma cells [12]. In addition to the previously highlighted resistance features observed in dormant cells from these two models, such as immune check-point expression (DA1-3b) or a stem cell-like phenotype (B16-F1) [11, 12], a deep investigation at genetic and nongenetic level was needed to unveil the intricate process of MRD. Moreover, we have previously shown shared key pathways between melanoma B16-F1 and leukemia DA1-3b models related to their aggressiveness features [13]. Thus, we proposed here to decipher mechanisms underlying MRD in these two distinct models to unveil common processes and therefore a potential “universal” tumor dormancy signature, regardless of tissue origin.

Using these two MRD models, we conducted a multiomics analysis including whole exome sequencing (WES) followed by targeted deep sequencing, copy number variation (CNV), chromatin immunoprecipitation followed by sequencing (ChIP-seq), transcriptomic and proteomic analysis. This approach aimed to elucidate the mechanisms underlying MRD in an immune system context, in order to characterize the tumour dormancy complexity and clarify how genetic and/or nongenetic events could play a role in MRD. This investigation marks the initial attempt to perform a multiomics approach based on dormant cells from syngeneic mouse models, therefore emphasizing specific genetic and nongenetic signatures. In this study, we revealed the potential impact of mutated genes trough their transcriptomic gene expression and involvement in signaling pathways. Regarding the proteomic signature of dormant cells, we identified dysregulated proteins involved in crucial pathways such as metabolism, differentiation and cytoskeleton reorganization. Although these two MRD models featured distinct signatures, 11 proteins were found to be commonly dysregulated in the melanoma and leukemia dormancy models. We also compared the murine MRD signature with corresponding human disease public datasets and revealed common features, reinforcing the translational potential of our MRD models.

## Results

### Identification of significantly mutated genes in murine models of dormant leukemia and melanoma

To determine whether therapeutic resistance arises from persistent or MRD cells through genetic mechanisms, we first compared the results of WES of dormant murine melanoma and leukemia (B16-F1GFP-D and DA1-3b/D365, respectively) cells to those of their parental cells (B16-F1GFP-M and DA1-3b). WES identified 218,914 variants in the whole samples, leading to 22,602 variants after filtering for quality and the normal genetic background. Sorting Intolerant From Tolerant (SIFT) algorithm prediction identified 190 significantly mutated genes (SMGs) (Additional file 1; Table S1-a). The SMGs identified in our mouse models included well-described melanoma and leukemia oncogenes and tumour suppressors (*Muc4*, *Pten*, *Grin2a*, *Dnmt3a*, *Npm1* and *Flt3*) [14–16] reinforcing the WES validation and the subsequent filtering of SMGs.

To validate SMGs and monitor gene harbouring mutation during MRD process, targeted sequencing was subsequently performed at various timepoints of the dormancy period in both models. In the murine leukemia model, targeted mutation monitoring was performed on Day 60 and Day 365 of dormancy (DA1-3b/D60 and DA1-3b/D365 cells respectively). Similarly, dormant melanoma B16-F1 cells were analysed on Day 365 of dormancy (B16-F1GFP-D) and during subsequent “generation” in a cell-derived brain site (B16-F1GFP-DB#1, #2, and #3 cells).

**Fig. 1:**
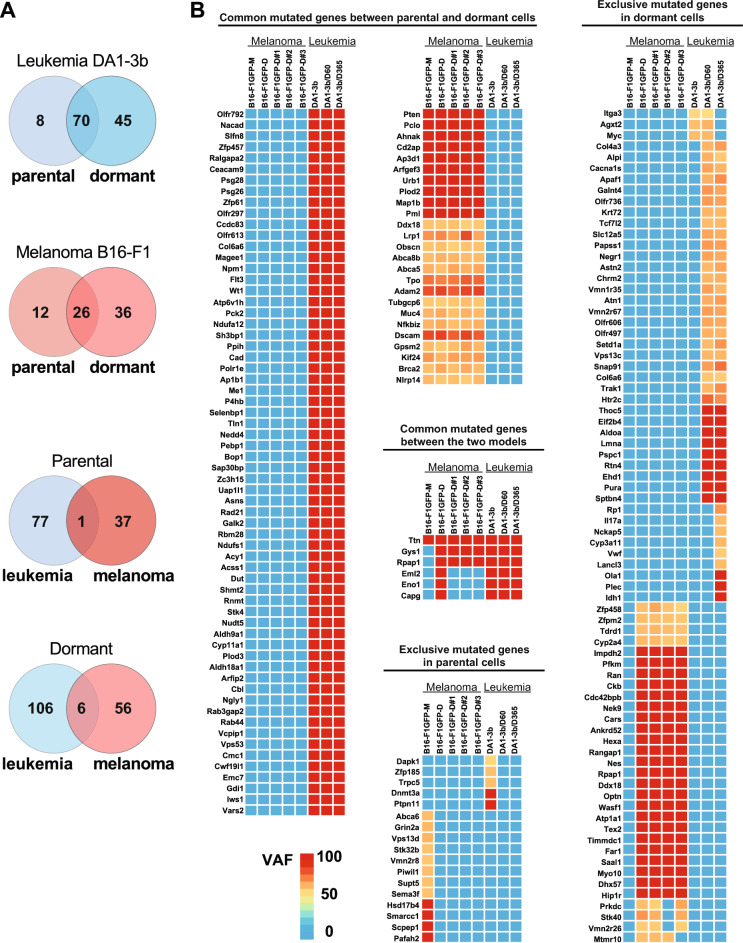
Genetic signatures of murine parental and dormant melanoma B16-F1 and leukemia DA1-3b cells. **A** Venn diagrams illustrating number of common or exclusively mutated genes according to the dormant state or the parental state or the tumour model. **B** Heatmaps showing genes bearing mutations in parental and dormant cells in melanoma B16-F1 and leukemia DA1-3b murine models. The colors in the heatmap correspond to the variant allelic frequency (VAF)

Through Venn diagrams, we visualized the number of shared or exclusively mutated genes based on the dormancy or parental state and the tumour type (Fig. 1A). Although a limited number of genes harbour mutations (six genes) overlapped between the melanoma and leukemia dormancy models (Fig. 1A, B), a notable number of mutations were exclusively found in dormant cells from both models (Fig. 1A, B).

In dormant DA1-3b/D365 cells, among the “dormancy” exclusive mutated genes, we observed 42 mutations potentially resulting in amino-acid (AA) changes. One frameshift mutation and three nonsense mutations were detected in the *Pcpc1* (M443X), *Tcf7l2* (R442*), *Chrm2* (Q358*) and *Atn1* (Q153*) genes. Regarding exclusively mutated genes in dormant melanoma B16-F1GFP-D cells, we observed 34 mutations that may lead to AA changes, while two nonsense mutations were observed in the *Cars* (W416*) and *Stk40* (Q386*) genes (Additional file 1, Table S1-a).

Specifically, approximately 48% *vs*. 6% of the targeted sequenced genes exclusively exhibited mutations in dormant versus parental melanoma cells, while in leukemia cells, this ratio was approximately 34% vs 17%. Despite identifying various common genes with mutations in both dormant and parental states within each model individually (Fig. 1B), signifying shared molecular characteristics, the MRD status might preferentially select clones with specific mutated genes (distinct between our 2 MRD models) conferring potential resistance against the microenvironment or therapies. The presence of exclusive gene mutations in parental cells in both models suggests the potential existence of the original dormant clones among the parental counterparts. On the other hand, exclusive gene mutations in dormant cells may indicate two potential scenarios: the acquisition of additional mutations during the MRD process and/or the existence of original mutations in preexisting dormant clones that were not technically detected by targeted sequencing among the heterogeneous parental clones.

To assess mutation clonality, Variant Allelic Frequency (VAF), serves as a metric of the representing the fraction of alleles carrying a specific genetic alteration, thus acting as a surrogate for mutation clonality [17]. Most mutations detected within dormant cells in both models exhibited high VAF values (> 50–100%), indicating that a substantial portion of cells harbour specific genetic alterations or mutations.

When we examined the genetic evolution of MRD, we found that dormant melanoma B16-F1GFP-D cells exhibited consistent genetic characteristics regardless of the dormancy/MRD duration. The genetic mutation signatures in the second generation of dormant melanoma cells (B16-F1GFP-DB#1, B16-F1GFP-DB#2, and B16-F1GFP-DB#3) were similar to those observed during the initial stage of dormancy analysis (B16-F1GFP-D). Conversely, in the murine leukemia model, subtle but noticeable differences in the number of gene mutations (nine genes) were identified between Day 60 and Day 365 of the dormancy process (Fig. 1B). From a genetic evolutionary perspective, it appears that MRD in the melanoma model could emerge from a pre-existing clone selection, whereas in the leukemia model, mutations may arise during the dormancy period.

### Multi-omics analysis of mutated genes revealed potential genetic implication for the dormancy phenotype in leukemia and melanoma models

**Fig. 2:**
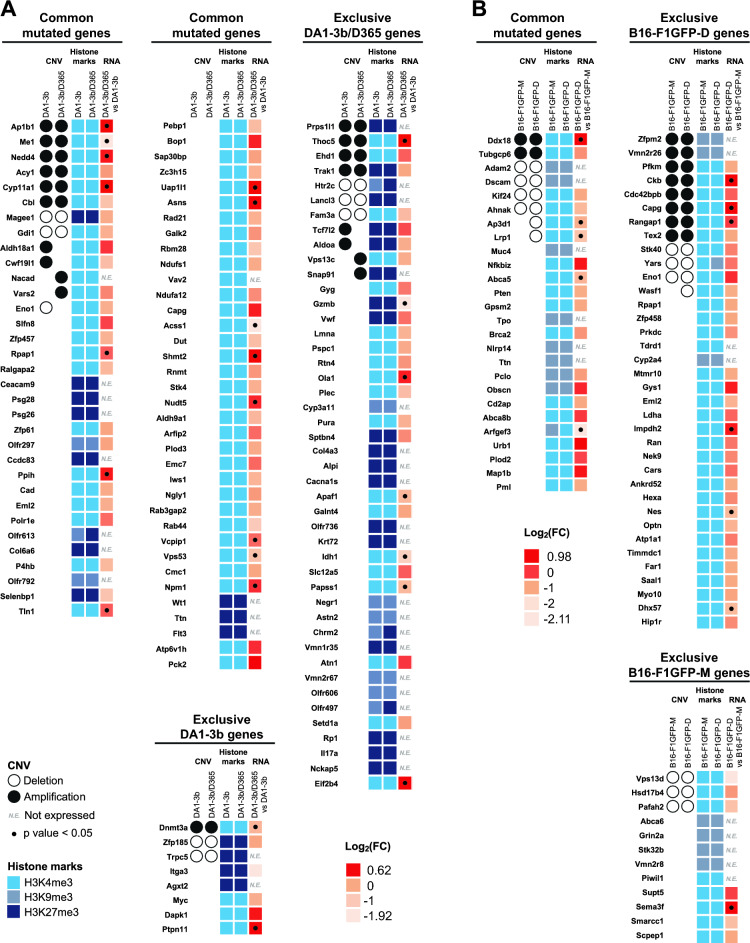
Heatmaps of the results of the CNV, histone marks, and transcriptomic gene expression analyses of MRD mutated genes. The CGH results are represented by a black circle for amplification, while deletions are denoted by an empty circle. H3K4me3 epigenetic active codes are depicted in bright blue, H3K9me3 in grey‒blue, and H3K27me3m in navy blue. Transcriptomic gene expression is depicted by a colour gradient relative to Log_2_ (fold change) varying from light orange for lower expression to red for higher expression. The fold change represents differential gene expression between dormant and parental cell conditions. Significant transcriptomic values (p < 0.05) are indicated with a dot. Each experiment was repeated three times. **A** Heatmap of the leukemia model DA1-3b. **B** Heatmap of the B16-F1 melanoma model

To better define the genetic signatures of the dormant cells from our two MRD models, we performed multiomics analysis and integrated the resulting CGH analysis (CNV), CHiP-seq (histones post-translational modifications), and transcriptomic gene expression data (Fig. 2, Additional file 1, Table S1-b).

Although the global CNV pattern at the arm level did not differ between dormant and parental cells in either model (Additional file 2, Fig.S1), several focal segments containing genes harbouring mutations were differentially amplified, deleted, or without any CNV alterations between the two conditions (parental and dormant status) (Additional file 1, Table S1-b).

We first analysed the DA1-3b leukemia model and among the 70 commonly mutated genes in dormant leukemia DA1-3b/D365 and parental DA1-3b cells, ten genes (*Ttc7b*, *Dnmt3b*, *Ap1b1*, *Ne1*, *Nedd4*, *Acy1*, *Cyp11a1*, *Htr2c*, *Magee1* and *Gdi1*) were amplified in dormant and parental cells (Fig. 2A). In addition, CNV loss was observed for *Magee1* and *Gdi1* regardless of the parental or dormant status of the cells. For 55 mutated genes, no amplification or deletion was noted in either dormant or parental leukemia cells. These observations highlighted the minimal impact of tumour dormancy on CNV for these indicated mutated genes.

In contrast, we identified CNV alterations of several mutated genes according to the dormant or parental status of the leukemic cells, signifying a specific genetic signature of dormant cells. For instance, *Aldh18a1* and *Cwf19l1* commonly mutated genes exhibited CNV amplification exclusively in the parental cells, while *Vars2* and *Eno1* showed exclusive amplification in the dormant cells and CNV loss in the parental cells respectively. Among the genes exclusively mutated in DA1-3b/D365 dormant cells, four genes (*Trak1*, *Prps1l1*, *Thoc5* and *Ehd1*) were amplified and three genes (*Lancl3*, *Fam3a* and *Htr2c*) shared by parental and dormant cells were deleted. Furthermore, the *Tcf7l2* and *Aldoa* genes displayed CNV amplification exclusively in the parental cells, while the *Vps13c* and *Snap91* genes were exclusively amplified in the dormant cells. Finally, for genes exclusively mutated in parental DA1-3b cells, two genes (*Zpf185* and *Trpc5*) exhibited deletions in both parental and dormant cells. Overall, integrative analysis of several commonly or exclusively mutated genes with differential CNV alterations according to the dormant or parental status of the leukemic cells allowed us to highlight/reveal genetic process (mutation pattern and CNV) for a subset of genes in the tumour dormancy or MRD context.

As we conducted WES to identify variants, except for mutations that lead to a nonsense AA, uncovered and filtered mutations were not found to be associated with regulatory sequences and therefore not directly linked to gene expression regulation. In contrast, the description of CNV signature of mutated genes in dormant leukemic cells may have a strong impact on gene expression.

To better understand the functional impact of the genetic signature (mutations and CNV) in the MRD process and uncover the regulatory mechanisms governing the expression of mutated genes, we performed additional analyses of epigenetic patterns combined with transcriptomic gene expression data. Regarding the epigenetic signature among mutated genes in the leukemia model, we focused on histone posttranslational modifications such as H3K4, H3K9, and H3K27 trimethylation (-me3) which reflect active (H3K4) and repressive (H3K9 and H3K27) gene transcription, respectively [18]. The greatest enrichment of H3K4me3 epigenetic modifications was observed for mutated genes shared by both parental and dormant cells. Among genes exclusively mutated in DA1-3b/D365 dormant cells, 40% (18 out of 45) were enriched with H3K27me3 modifications, a pattern conserved in both dormant and parental cells. In contrast, for genes that were exclusively mutated in the parental DA1-3b cells, 50% (four out of eight) displayed H3K27me3 repressive histone marks, while the remaining genes exhibited H3K4me3 active pattern. As histone marks are closely related to the regulation of gene expression, we examined transcriptomic gene expression and observed its variability among mutated genes mainly according to the corresponding epigenetic pattern. As expected, 26 out of 36 and seven out of 11 mutated genes enriched with H3K27me3 and H3K9me3 repressive epigenetic code, respectively, displayed no RNA transcription, while only two out of 81 mutated genes enriched with H3K4me3 active marks showed no transcriptomic gene expression (Fig. 2A).

Notably, in the DA1-3b/D365 dormant cells, the mutated genes whose expression was most strongly compared to that in the parental cells, included *Nudt5*,* Shmt2*,* Asns*,* Pck2*,* Eno1*,* Npm1*, and *Bop1*, with the first five genes known for their involvement in metabolic pathways. *Thoc5, Eif2b4*, and *Ola1* overexpressing mutated genes were found exclusively in DA1-3b/D365 dormant cells. In contrast, among genes exclusively mutated in the DA1-3b parental cells, *Ptpn11 *and *Dapk1* were the most highly expressed genes (Fig. 2A). Although the expression of mutated genes may have an impact without overexpression, these upregulated mutated genes were predominantly not related to CNV gain or loss pattern differences highlighting a further complex mechanism or combined nongenetic and genetic mechanisms contributing to the MRD phenomenon.

A similar multiomics approach was conducted with the identified mutated genes in the murine melanoma MRD model. Among the 26 shared mutated genes in melanoma B16-F1GFP-D and B16-FGFP-M cells, two genes (*Ddx18* and *Tubgcp6*) were amplified in both parental and dormant cells (Fig. 2B). CNV loss was observed for the *Adam2, Dscam, Kif24* and *Ahnak* genes in both parental and dormant cells. No genomic alterations were revealed in either dormant or parental melanoma cells for 18 commonly mutated genes including oncogenes or tumour suppressor genes such as *Pten*, *Brca2*, or* Pml*. As observed for the leukemia MRD model, our results revealed a minimal impact of melanoma dormancy on CNV for these particular mutated genes.

Conversely, the two *Lrp1* and *Ap3d1* mutated genes shared by in both dormant and parental cells, exhibited CNV deletions exclusively in dormant cells. Among genes exclusively mutated in B16-F1GFP-D dormant cells, eight genes (*Zpfm2*,* Vmn2r26*,* Pfkm*,* Ckb*,* Cdc42bpb*,* Capg*,* Rangap1* and *Tex2*) were amplified, and three genes (*Stk40*,* Yars* and *Eno1*) shared by parental and dormant cells were deleted. In addition, *Wasp1* displayed CNV deletion exclusively in dormant cells. With respect to genes that were exclusively mutated in the parental B16-F1GFP-M cells, three genes (*Vps13d*,* Hsd17b4* and *Pafah2*) exhibited deletions in both the parental and dormant cells. As observed in the leukemia model, identification of several mutated genes combined with differential CNV alterations according to the dormant or parental status highlighted genetic involvement underlying the MRD context.

We next adopted a similar strategy for the melanoma model and performed additional analysis regarding epigenetic patterns combined with transcriptomic gene expression. The greatest enrichment of H3K4me3 epigenetic modifications was observed with 17 mutated genes shared by both parental and dormant cells. Among the genes exclusively mutated in the B16-FGFP-D dormant cells, 8% (three out of 36) were enriched with H3K9me3 modifications, a pattern conserved in both cell lines. In contrast, for genes exclusively mutated in parental DA1-3b cells, 30% (four out of 13) displayed H3K9me3 repressive histone codes, while the remaining genes exhibited H3K4me3 active marks. As expected, 13 out of 15 mutated genes enriched with the H3K9me3 repressive epigenetic code demonstrated no transcriptomic gene expression. In comparison, only one out of 56 mutated genes enriched with the H3K4me3 active mark were not detected (Fig. 2A).

Overall, although the global CNV and/or epigenetic profiles were essentially identical between dormant and parental cells in both leukemia and melanoma models, the identification of specific mutated gene signatures in dormant cells associated with a differential CNV pattern can highlight the genetic mechanisms underlying the MRD phenomenon. The functional impact of the identified mutated genes and/or the regulation of their expression may shape the dormant cell phenotype in both MRD models. Despite the fact that tumour dormancy involved equivalent genetic implication in both models, i.e., not identical mutated genes but rather similar multiomics signature, we observed a significant difference in the percentage of mutated gene exclusively expressed in dormant cells: 89% vs. 56% in leukemia and melanoma respectively.

### Mutated genes were involved in signalling pathways

In our study models, we first defined GO based on the molecular functions, biological pathways, and cytological components associated with gene products. By conducting GO enrichment analysis, we aimed to elucidate how genes with mutations in both parental and dormant cells in the MRD models are functionally linked.

**Fig. 3:**
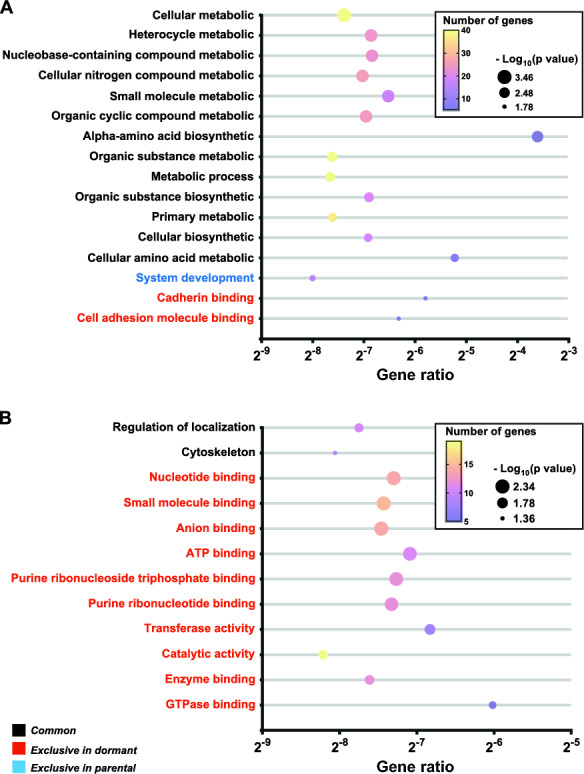
Bubble plots of pathway enrichment analysis with MRD mutated genes in the DA1-3b (**A**) and B16-F1 (**B**) models. Pathways are classified (colours black, red and blue) according to the common and exclusive mutated genes in the dormant and parental cells. The number of genes indicates the number of mutated genes enriched in the pathway (colour plasma gradient). The “Gene Ratio” indicates the ratio of enriched mutated genes to background genes. Bubble size is according to the p value (−Log_10_ scale) of pathway enrichment

Our results revealed 12 significantly enriched terms for commonly mutated genes in parental leukemia cells and two in parental melanoma cells. Additionally, only genes with mutations in dormant cells were significantly enriched in two and nine pathways in the DA1-3b/D365 and B16-F1GFP-D models, respectively. Interestingly, no enrichment was observed among exclusively mutated genes in melanoma parental cells, except for one significant GO term in leukemia DA1-3b counterpart cells (Fig. 3A, B).

The exclusively mutated genes in DA1-3b/D365 cells were enriched in biological functions such as “cadherin binding” and “cell adhesion molecule binding”. The exclusively mutated genes in B16-F1GFP-D were enriched in various biological functions, including “small molecule binding,” “nucleotide binding,” “catalytic activity,” and “enzyme binding”.

### Genetic profiles of the “murine MRD mutated gene signature” in human leukemia and melanoma diseases

**Fig. 4:**
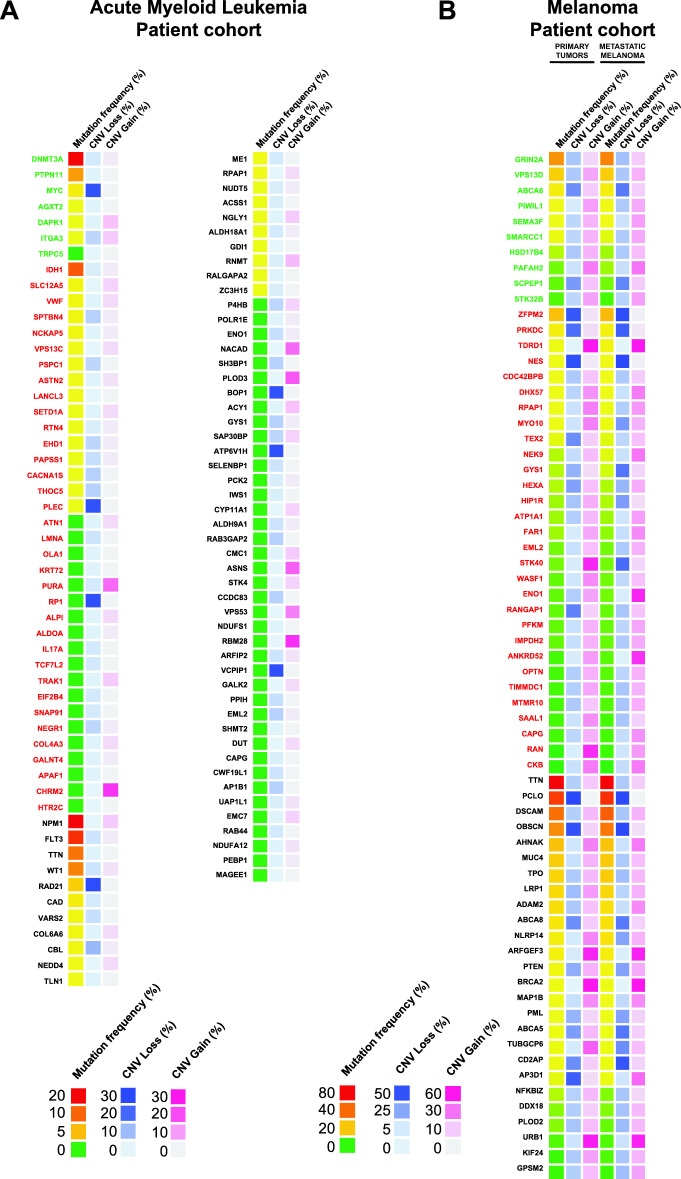
Heatmaps of “MRD genes” corresponding to genetic profiles in human AML (**A**) and melanoma (**B**) patients. The frequencies of mutated genes and associated CNVs (loss and gain) are expressed as percentages and reflect the number of patients affected among the total patients in the corresponding AML and melanoma patient cohorts (n = 207, n = 489 for AML and melanoma, respectively). The values are represented by a distinct colour gradient. For human melanoma samples, the results are represented according to the stage of disease progression, i.e., primary or metastatic tumours. The data were extracted from the public domain GDC portal (NIH Institute) version 1.0. In the indicated list, commonly mutated genes are indicated in black letters, genes exclusive to dormant cells are shown in red letters, and genes exclusive to parental cells are displayed in green letters

To determine the possible implication in human disease of the mutated genes through our murine MRD models, we analysed the frequency of mutations in these in human acute myeloid leukemia (AML) and primary and metastatic melanoma tumours. The results were extracted from several patient cohorts (n = 207 for AML patients and n = 489 for melanoma patients) in public datasets (GDC portal) that combined exome sequencing data. The frequencies of mutated genes in human samples among the genes found to be mutated in murine models of leukemia (Fig. 4A) or melanoma (Fig. 4B) were noted (Additional file 1, Table S1-c).

Only data corresponding to the diagnosis of AML were documented. Among the genes exclusively mutated in the dormant model, only the *IDH1* gene was highly frequently mutated (10.63%) in human AML samples while the remaining genes were mutated either at a low frequency (under 1.5%) or not at all (Fig. 4A). Although several genes (40 genes) identified among commonly mutated genes in murine dormant and parental cells were not genetically affected in human AML, a substantial number of genes (21 genes) displayed mutations, including 6 genes with moderate to high frequencies. As expected, the most frequently mutated genes were *DNMT3A* (19.32%) and *NPM1* (20.29%), followed by *FLT3* (13.53%) which is consistent with the AML mutation landscape [14] as shown in Additional file 1, Table S1-d, reinforcing the relevance of our DA1-3b leukemia model for future translational applications in humans. Additionally, *PTPN11* (6.76%) was one of the most frequently mutated genes in human AML samples and was also exclusively mutated in the murine parental model. In the literature, *PTPN11* is notably described for its significant role in leukemia development, where its mutations can lead to hyperactivation, causing proliferation of leukemic cells, therapy resistance, and survival [19].

Interestingly, despite the lack of mirroring regarding mutations in human AML, CNV analysis revealed that several genes mutated exclusively in dormant cells or under both conditions, were either amplified or deleted in human AML. Indeed, *PURA* (16.35%), *CHMR2* (23.08%), *NACAD* (16.35%), *PLOD3* (18.27%), *ASNS* (17.31%), *VPS53* (13.46%), and *RBM28* (24.04%) were particularly altered with CNV gain. Conversely, the *PLEC* (25.96%), *RP1* (26.92%), *RAD21* (27.88%), *BOP1* (25.96%), *ATP6V1H* (26.92%), and *VCPIP1* (26.92%) genes were strongly associated with CNV loss (Fig. 4A). Interestingly, the uncovered CNV alterations of these particular genes in human AML patients even at the time of diagnosis reinforced the impact of these “dormancy” gene signatures.

We next explored the mutation frequencies of “murine MRD genes” in human melanoma samples across all conditions, including primary tumour and metastatic stages (Fig. 4B). Overall, a similar trend was observed regardless of the condition (primary tumours, and metastatic melanoma). The most frequently mutated genes in human melanoma were those mutated in parental and dormant mouse cells (common genes), with up to 80% mutation rates for *TTN*, followed by *PCLO* (> 52%), *DSCAM* (approximately 40%), and *OBSCN* (approximately 34%). *GRIN2A* followed among genes exclusive to the parental cells (approximately 32%). These genes are frequently mutated in human melanoma (Fig. S2), reinforcing the relevance of our B16-F1 murine model. Interestingly, several exclusively mutated genes in murine dormant cells such as *ZFPM2*, *PRKDC*, *TDRD1*, *NES*, *CDC42BPBD* and *HX57*, exhibited moderate to high frequencies of mutation in human melanoma.

Although the mutation frequency did not vary according to disease stage, the results were largely the same for CNV gains and losses. However, a substantial number of genes appeared to stand out. In the context of CNV gain, exclusively mutated genes in dormant cells such as *TEX2* were associated with a decrease in metastasis, as was *RANGAP1* (lack of 15%). Conversely, *STK40* showed an increase in metastasis (30.5%). Commonly mutated genes, such as *AP3D1*, demonstrated a pronounced disparity, with a significantly greater percentage observed in primary tumours (38% vs. 8% in metastatic stage). In the context of CNV loss, the analysis revealed notable patterns for “murine MRD genes”. *STK40* exhibited a disparity, as it increased in primary tumours (53.08%), such as *RAN* (49.66%). In contrast, *ENO1* showed a substantial increase (51.72%) in metastases, as did *ANKRD52* (47.48%). These findings underscore the diversity of CNV gains and losses in “murine MRD genes”.

Even though analysis at the metastatic stage does not perfectly mimic the MRD signature in human melanoma, it helps to highlight genetic patterns of cells that have survived to anticancer treatment and/or in different microenvironments compared to those of primary tumours. Although the “murine MRD genes” mutation signature was not clearly enriched in human melanoma metastasis, this analysis revealed that for a few genes, the associated CNV alterations were correlated with the metastatic stage of the disease.

### Differential protein expression in dormant leukemia DA1-3b/D365 cells sheds light on a distinct dominant nongenetic process in MRD

As the functional impact of the genetic signature reflected by expression of mutated genes could not define the exclusively dormant cell phenotype, we next conducted proteomic signature analysis of dormant leukemic cells to uncover potential differential and exclusive protein expression. We questioned which proteins were dysregulated in dormant cells and if identified mutated genes were expressed at the protein level and/or overlapped with the differentially proteins expressed.

**Fig. 5:**
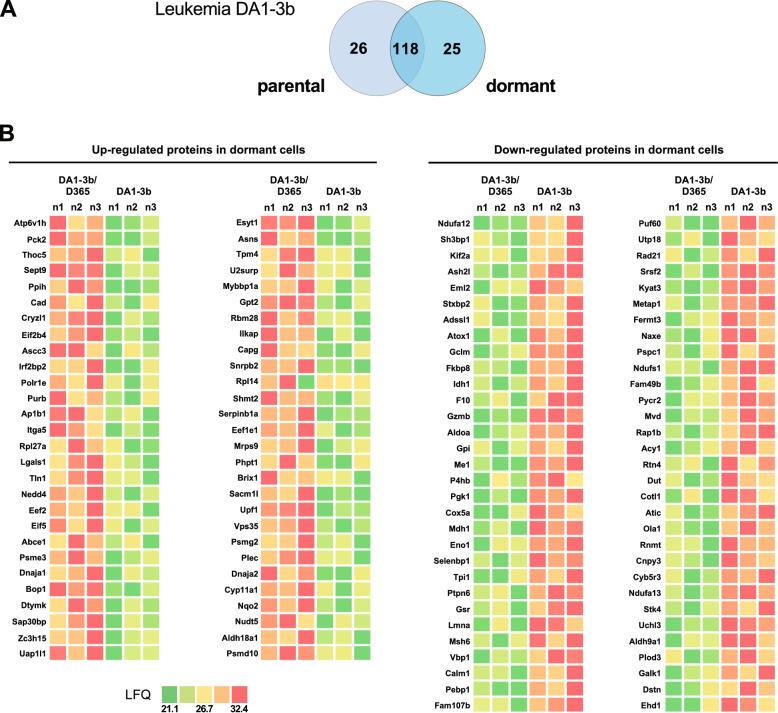
Differential protein expression analysis in dormant leukemia DA1-3b cells compared to their parental cells. **A** Venn diagrams illustrating the number of commonly dysregulated and exclusive proteins according to the dormant or parental state of leukemia DA1-3b cells. A significance threshold of P < 0.05 was used for Student’s t test with Perseus software. **B** Heatmaps illustrating upregulated (red colour gradient) and downregulated proteins (green colour gradient) in leukemia DA1-3b/D365 cells compared to their parental cells. The results are expressed as label-free quantification (LFQ) of proteins. The LFQ intensity was logarithmized (log2[x]). Three independent experiments (n = 3) were performed, and the resulting LFQs are shown

Hence, mass spectrometry (MS) was used to analyse protein expression variations in dormant leukemia DA-1b/D365 cells. A total of 2,182 proteins were identified, with 118 proteins displaying significant differences in expression levels (p < 0.01) between DA1-3b/D365 and DA1-3b cells. Notably, 26 proteins were uniquely expressed in parental cells, while 25 proteins were exclusively detected in dormant cells (Fig. 5A) (Additional file 1, Table S1-e). Among 118 proteins with altered expression, 56 were upregulated, whereas 63 were downregulated specifically in dormant cells (Fig. 5B) (Additional file 1, Table S1-e).

To better define the proteomic signature of dormant leukemic cells, we conducted multiomics analysis combining CGH, epigenetic modification and transcriptomic gene expression data. Our objective was to determine how genetic or nongenetic mechanisms shape underlying mechanisms that lead to dormant signature/phenotype.

**Fig. 6:**
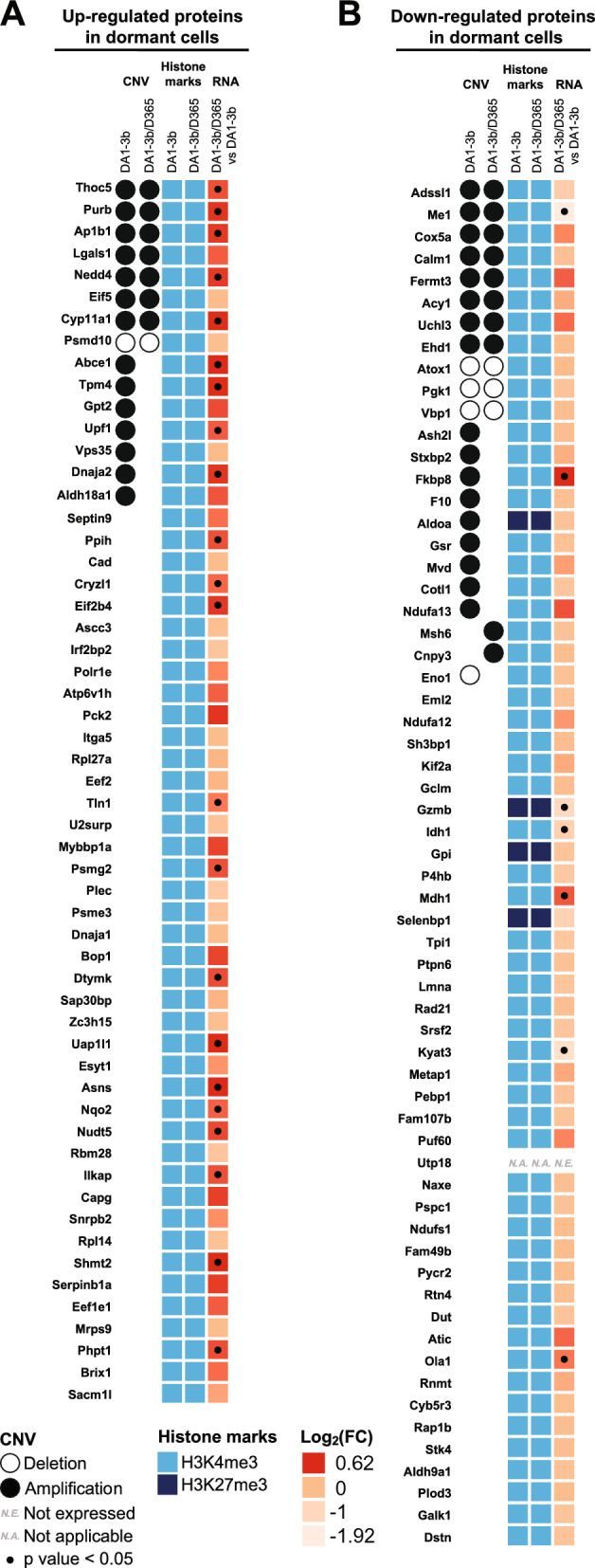
Heatmaps of the results of CNV, histone marks and transcriptomic gene expression of dysregulated proteins in dormant leukemic DA1-3b/D365 cells compared to their parental cells. CGH results are represented by a black circle for amplification, while deletions are denoted by an empty circle. H3K4me3 epigenetic active codes are depicted in bright blue and H3K27me3 in navy blue. Transcriptomic gene expression is depicted by a colour gradient relative to Log_2_ (fold change) varying from light orange for lower expression to red for higher expression. The fold change represents differential gene expression between dormant and parental cell conditions. Significant transcriptomic values (p < 0.05) are indicated with a dot. Each experiment was repeated three times. **A** Heatmap of the upregulated proteins in dormant cells. **B** Heatmap of the downregulated proteins in dormant cells

Among the upregulated proteins in the dormant cells, seven out of 56 genes showed amplification in both the parental and dormant cells, while seven genes were exclusively amplified in the parental cells. The remaining 42 corresponding genes were neither amplified nor deleted. Additionally, only *Psdm10* exhibited a deletion in both parental and dormant cells. Regarding proteins downregulated in dormant cells, eight proteins displayed amplification in both cell lines, with nine exclusively amplified in parental cells and two solely in the dormant cells. The remaining 53 corresponding genes displayed no genomic alterations. Except for the nine exclusively amplified genes in parental cells, the CNV differential pattern between dormant and parental cells did not explain the differential protein expression. These observations highlighted the possible nongenetic mechanisms underlying the dysregulated proteomic signature of dormant leukemic cells (Fig. 6A).

We next conducted epigenetic modification analysis and H3K4me3, H3K9me3, and H3K27me3 epigenetic marks consistently revealed a pronounced prevalence of H3K4me3 modification in dormant and parental cells, with only four showing enrichment of the H3K27me3 repressive code across all genes. Among the proteins that were upregulated in dormant cells, 22 showed significantly increased RNA transcription. Specifically, among the upregulated proteins showing significantly increased transcriptomic expression in dormant cells, the following proteins were notable: THOC5, PPIH, CRYZL1, PURB, AP1B1, TTN1, NEDD4, ABCE1, DTYMK, UAP1L1, ASNS, TPM4, ILKAP, SHMT2, PHPT1, UPF1, PSMG2, DNAJA2, CYP11A1, NQO2 and NUDT5. Among these proteins, DNAJA2, ASNS, SHMT2, DTYMK, and NUDT5 are involved in acetylation mechanisms influencing protein structure and gene expression. Additionally, DNAJA2, ASNS, DTYMK, NEDD4, ABCE1, and UPF1 are known for their involvement in cytosolic processes (Fig. 6A). Conversely, among the proteins downregulated in dormant cells, seven showed a significant difference in transcriptomic gene expression. Three proteins exhibited deletions in both dormant and parental cells, with *Eno1* being deleted solely in the parental cells (Fig. 6B).

Proteins exclusively detected in the DA1-3b/D365 dormant cells displayed amplification of three corresponding genes in both the parental and dormant cells; four were exclusively amplified in the parental cells, and only *Ass1* was amplified exclusively in dormant cells. For proteins exclusively expressed in the DA1-3b parental model, three were amplified only in the parental cells, while *Vars2* was amplified solely in the dormant cells. Furthermore, three genes exhibited deletions in parental and dormant cells. Notably, only *F13a1* exhibited a differential epigenetic pattern between the parental cells, marked by H3K9me3, and the dormant cells, characterized by H3K27me3. The transcriptomic expression of genes identified through proteomic analysis in the leukemia model indicated a generally uniform expression across all proteins, with notable overexpression observed for *Ass1*, which was exclusive to the DA1-3b/D365 dormant cells (Fig S2).

Overall, our results showed that the dysregulated proteins in dormant leukemic cells were mainly unrelated to genetic/genomic alterations. In addition, except for a discrete number of genes, the identified mutated genes were not differentially expressed at the protein level.

### Differential protein expression in a murine leukemia model highlights its involvement in biological pathways

**Fig. 7:**
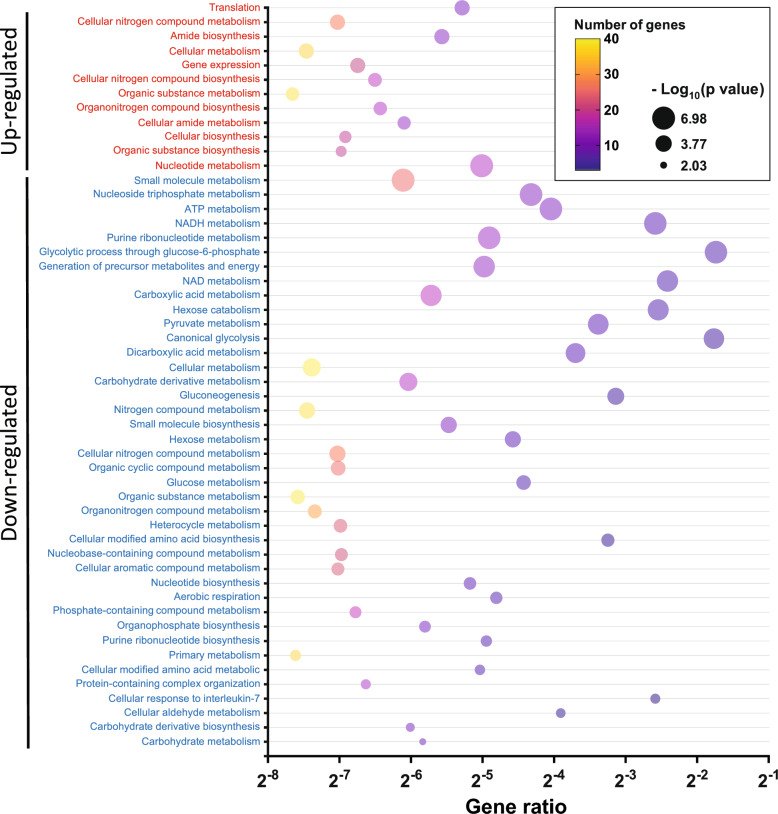
Bubble plots of GO pathway enrichment analysis of differentially expressed proteins in dormant leukemia DA1-3b/D365 cells compared to their parental cells. Pathways are classified according to upregulated (red) and downregulated (blue) protein expression. The number of proteins enriched in the pathway is indicated (colour plasma gradient). The “Gene Ratio” indicates the ratio of enriched proteins to background proteins. Bubble size is according to the p value (Log_10_ scale) of pathway enrichment

As shown in Fig. 7, a bubble plot demonstrates our GO enrichment analysis aiming to uncover the functional roles of proteins differentially expressed in dormant DA1-3B/D365 cells compared to their parental counterparts. This analysis revealed 12 significantly enriched terms related to the upregulated proteins, primarily related to “cellular metabolism” and “organo-nitrogen compound biosynthesis.” Notably, the GO enrichment analysis highlighted pathways with the highest gene ratio, specifically emphasizing “translation,” “amide biosynthesis,” and “cellular amide metabolism.”

Conversely, the downregulated proteins were enriched in 40 distinct terms, with a particular emphasis on processes such as “glycolytic process through glucose-6-phosphate” and “canonical glycolysis.” The pathways exhibiting the highest gene ratios were those linked to “cellular metabolism,” “nitrogen compound metabolism,” and “cellular modified amino acid biosynthesis” as illustrated in Fig. 7. These discoveries offer valuable insights into the underlying molecular mechanisms governing the behaviour of dormant leukemia cells and their original counterparts.

### Differential protein expression analysis in dormant melanoma B16-F1GFP-D cells

**Fig. 8:**
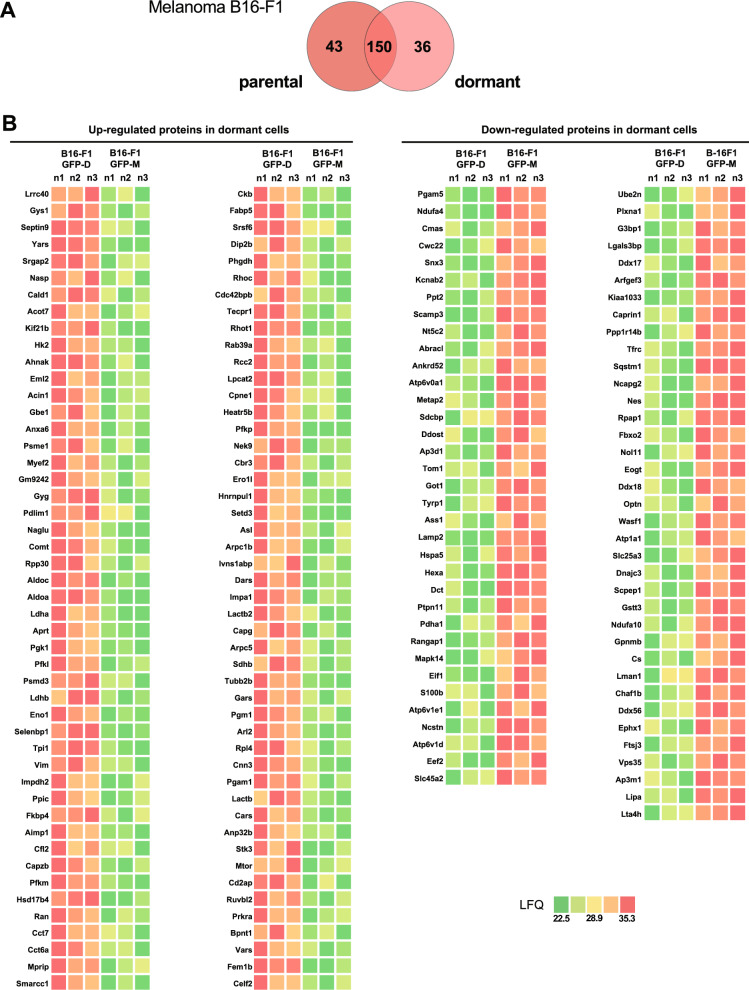
Differential protein expression analysis in dormant melanoma B16-F1GFP-D cells compared to their parental cells. **A** Venn diagrams illustrating the number of common dysregulated and exclusive proteins according to the dormant or parental state of the melanoma B16-F1 cells. A significance threshold of p < 0.05 was used for Student’s t test with Perseus software. **B** Heatmaps illustrating upregulated (red colour gradient) and downregulated proteins (green colour gradient) in dormant melanoma B16-F1GFP-D cells compared to their parental counterpart cells. The results are expressed as label-free quantification (LFQ) of proteins. The LFQ intensity was logarithmized (log2[x]). Three independent experiments (n = 3) were performed, and the resulting LFQs are shown

Analysis of protein expression variations in dormant melanoma B16-F1GFP-D cells was also performed through proteomic investigation using mass spectrometry. A total of 2474 proteins were identified, 168 of which exhibited differences in expression (p < 0.05) between B16-F1GFP-D and B16-F1GFP-M cells. Notably, 43 proteins were only expressed in parental cells, while 36 proteins were exclusively detected in dormant cells (Fig. 8A). Among the 168 proteins whose expression was altered, 96 were upregulated, whereas 72 were downregulated in the dormant melanoma B16-F1GFP-D cells (Fig. 8B, Additional file 1, Table S1-f).

**Fig. 9:**
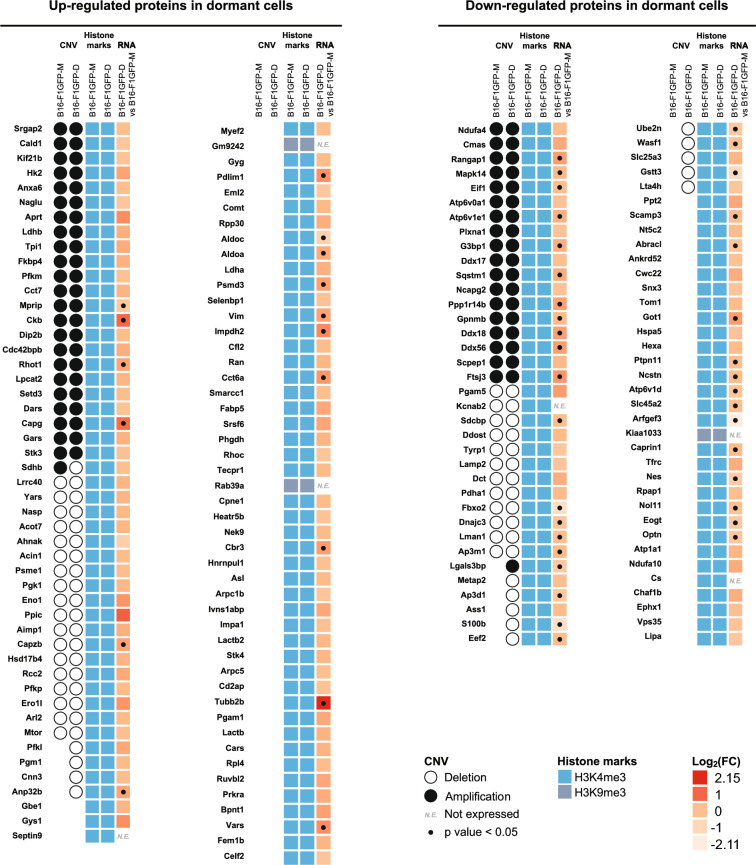
Heatmaps of the results of CNV, histone marks and transcriptomic gene expression of dysregulated proteins in dormant melanoma B16-F1GFP-D cells compared to their parental cells. CGH results are represented by a black circle for amplification, while deletions are denoted by an empty circle. H3K4me3 epigenetic active codes are depicted in bright blue and H3K9me3 in grey‒blue.. Transcriptomic gene expression is depicted by a colour gradient relative to Log_2_ (fold change) varying from light orange for lower expression to red for higher expression. The fold change represents differential gene expression between dormant and parental cell conditions. Significant transcriptomic values (p < 0.05) are indicated with dots. Each experiment was repeated three times

As performed in the leukemia model, we used multiomics approaches to integrate transcriptomic, CGH-based detection of CNV regions, and epigenetic modification analysis of the identified proteins (Fig. 9, Additional file 1, Table S1-g). As expected, most of the corresponding genes exhibited an H3K4me3 epigenetic active mark, i.e., 94 out of 96 upregulated genes and 69 out of 70 downregulated proteins indicating an active transcription. Additionally, CGH analysis revealed that for the majority of genes encoding proteins, 42 upregulated and 30 downregulated proteins, exhibited amplification or deletion patterns conserved between parental and dormant cells. Although we observed no correlation between the differential CNV pattern and the upregulation of proteins in dormant cells, deletions specifically in the dormant model were observed for nine proteins (METAP2, ASS1, S100B, EEF2, UBE2N, WASF1, SLC25A3, GSTT3 and LTA4H) of the downregulated ones, suggesting that tumour dormancy may impact the expression of these genes through genomic alteration.

Regarding proteins exclusively detected in dormant cells, no differential CNV pattern correlated with proteomic analysis (Additional file 3, Fig. S2). Interestingly, we detected high transcriptomic expression of *Adssl1* in dormant cells without any genomic variation. Among proteins exclusively detected in parental cells, most of the genes displayed no differential CNV pattern between dormant and parental cells except for the *Stx7* and *Wdr18* genes which exhibited exclusive deletions in dormant cells (Additional file 3, Fig. S2).

In summary, our results revealed that protein dysregulation in dormant melanoma cells largely did not occur through genetic mechanisms, in concordance with what was observed in the leukemia MRD model. Furthermore, apart from a few genes, the identified mutated genes did not exhibit differential expression at the protein level.

### Differential protein expression in a murine melanoma model highlights the significant involvement of biological pathways

**Fig. 10:**
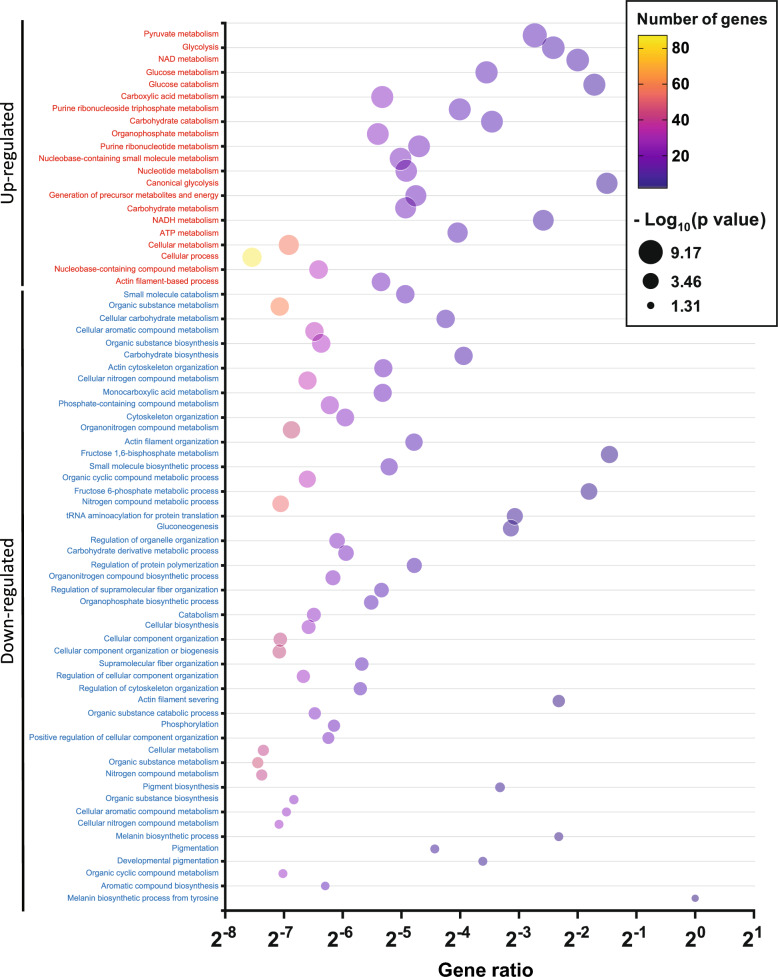
Bubble plots of GO pathway enrichment analysis of differentially expressed proteins in dormant melanoma B16-F1 cells compared to their parental cells. Pathways are classified according to upregulated (red) and downregulated (blue) protein expression. The number of proteins enriched in each pathway is indicated by the colour of the plasma gradient. The “Gene Ratio” indicates the ratio of enriched proteins to background proteins. Bubble size is according to the p value (Log_10_ scale) of pathway enrichment

The subsequent step involved conducting GO enrichment analysis to explore the functional relationships of differentially expressed proteins in dormant B16-F1GFP-D cells compared to their parental counterparts. A total of 21 terms exhibited significant enrichment for upregulated proteins, primarily associated with cellular processes and metabolism, particularly involving NAD metabolism, glucose catabolism, and canonical glycolysis pathways (Fig. 10). Conversely, downregulated proteins were enriched in 51 terms, with a focus on various metabolic processes, cellular component organization, and biogenesis. The key pathways identified included proton transmembrane transport, actin filament severing, melanin biosynthesis, fructose metabolism, and cellular component biogenesis, as depicted in Fig. 10.

### Identification of 11 common differentially expressed genes in both murine MRD models revealed a possible common MRD signature

**Fig. 11:**
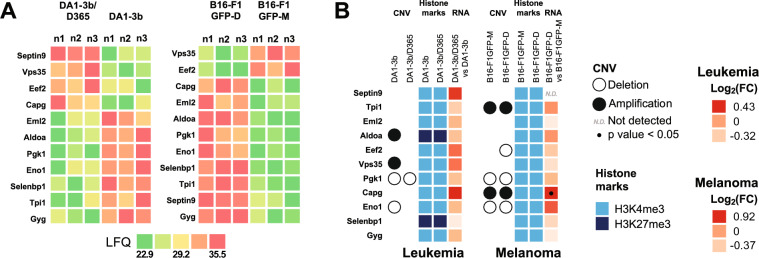
Common protein signatures between the 2 murine MRD models. **A** Heatmaps illustrating upregulated and downregulated proteins in dormant melanoma B16-F1-GFP-D and leukemia DA1-3b/D365 cells compared to their parental cells. The results are expressed as label-free quantification (LFQ) of proteins. The LFQ intensity was logarithmized (log2[x]). Three independent experiments (n = 3) were performed, and the resulting LFQs are shown. **B** Heatmaps of integrative data analysis of CNV, histone marks and transcriptomic gene expression with dysregulated proteins in dormant melanoma B16-F1GFP-D and leukemia DA1-3b/D365 cells compared to their parental cells**.** CGH results are represented by a black circle for amplification, while deletions are denoted by an empty circle. H3K4me3 active marks are depicted in bright blue and H3K27me3 in navy blue. Transcriptomic gene expression is depicted by a colour gradient relative to Log_2_ (fold change) varying from light orange for lower expression to red for higher expression. The fold change represents differential gene expression between dormant and parental cell conditions. Significant transcriptomic values (p < 0.05) are indicated with a dot. Each experiment was repeated three times

Among the 11 proteins commonly differentially expressed between the leukemia and melanoma models, four exhibited increased expression in the leukemia dormancy model. In contrast, nine proteins showed increased expression in the melanoma dormancy model. Notably, only SEPTIN-9 and CAPG exhibited consistent overexpression in both pathologies; this suggests a distinct protein signature between the two conditions (Fig. 11A).

Among these 11 proteins, CAPG is commonly overexpressed in both leukemia and melanoma dormant cells. SEPTIN9 is known for its role in regulating cell structure and cell division, while CAPG influences cell motility and shape by modulating actin dynamics. These two proteins are therefore crucial for many fundamental cellular processes. Their observed increase in protein expression in the dormancy models of the two pathologies suggests a potential structural alteration of the cytoskeleton in the context of tumour dormancy. Furthermore, methylation of the *Septin9* promoter has recently been associated with cancer recurrence and metastasis phenomena, particularly in breast cancer [20]. The role of CAPG in AML promotion has been described [21] (Fig. 11B).

The comparative analysis of CNVs in both models revealed a disparity in protein amplification. However, a shared deletion between the parental and dormant cells was observed in both pathologies, specifically involving *Pgk1*. Additionally, the parental deletion of *Eno1* is consistent across the parental model of melanoma and its associated dormancy model. As expected, we observed a predominance of the H3K4me3 epigenetic active pattern in both pathologies; however, the H3K27me3 repressive histone code was detected for the *Aldoa* and *Selenbp1* genes in the leukemic model (Fig. 11B, additional file 1, Table S1-h).

### Genetic and differential expression impact of the 11-protein MRD signature in human AML and melanoma

Among the 11 proteins commonly dysregulated in dormant cells from both pathologies (Table 1), public data analysis from AML patient cohorts [22, 23] (n = 905) revealed that four of them—*SELENBP1*, *SEPTIN9*, *GYG*, and *ENO1*—demonstrated significantly greater expression in patients with adverse prognoses (ELN 2017 classification). Conversely, the remaining proteins appeared to be associated with an intermediate prognosis. Notably, a discrete percentage (0.5%) of patients displaying CNV (deletion) was observed in four of these common genes, including *ENO1*, which also exhibited a deletion in our murine parental myeloid model. Regarding melanoma pathology, when comparing public data from patients between the diagnosis and metastatic stages [16], significant frequencies of amplification (14.5% and 10.0% respectively) were observed for the *SELENBP1* and *SEPTIN9* genes at the metastatic stage (Table 1). Consistent with findings from our parental and dormant mouse melanoma models, a notable frequency of *TPI1* amplification at both the diagnostic (4.7%) and metastatic (6.4%) stages was observed.

**Table 1:** Impact of the 11 proteins commonly differentially expressed in murine DA1-3b/D365 leukemia and B16-F1GFP-D melanoma dormancy models on human AML and melanoma

Gene	Acute myeloid leukemia		Melanoma—primary tumours		Metastatic melanoma	
	ENL classification (higher expression in)		CNV loss and gain frequency (%)			
		Deletion	Amplification	Deletion	Amplification	Deletion
ALDOA	Intermediate*	0.5	4.7	1.6	2.7	
SELENBP1	Adverse*		6.3		14.5	0.9
SEPTIN9	Adverse*	0.5	7.8	1.6	10.0	
EEF2	Intermediate*					
CAPG	Intermediate*		6.3	1.6	0.9	0.9
EML2	Intermediate				4.5	2.7
GYG	Adverse		4.7		1.8	
VPS35	Intermediate	0.5	1.6	3.1		0.9
TPI1	Intermediate		4.7		6.4	6.4
ENO1	Adverse	0.5	4.7	6.3	1.8	1.8
PGK1	Intermediate					

Table 1 reveals a correlation between the highest expression (RNA-seq) of the 11 genes and ELN classification groups (i.e. adverse, intermediate and favourable) in AML (n = 905). An asterisk symbolizes a significant result (p < 0.05). The table shows the frequency (%) of CNV loss and gain related to the indicated gene in AML and melanoma (primary and metastatic tumors) patients (n = 489). The data were extracted from the public domain cBioportal [16, 22, 23].

### Shared functional properties by dormant cells from the both MRD models

**Fig. 12:**
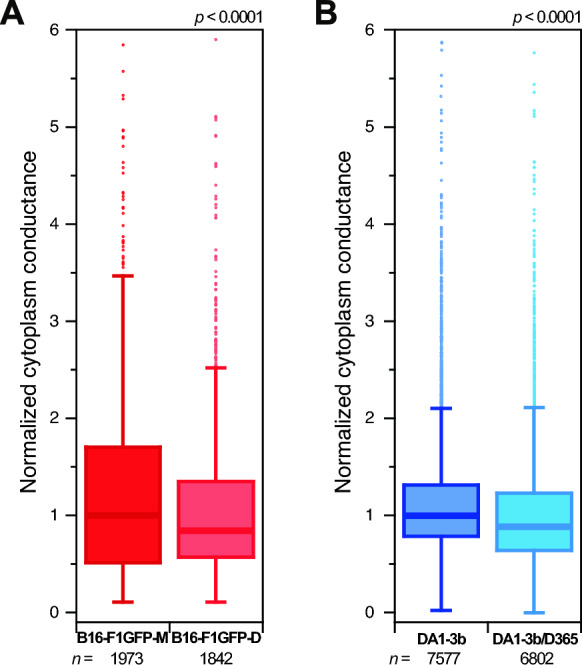
Box plots comparing the cytoplasm conductance of both dormant melanoma B16-F1GFP-D cells and dormant leukemia DA1-3b/D365 cells compared to their parental counterpart cells. Cells of each group, **A** melanoma and **B** leukemia, were analysed by impedance measurements at 30 MHz, and normalized according to the median of parental cells. The experiments were repeated four and six times, respectively, with independent cell cultures. The numbers of analysed cells are shown under each plot. Both comparisons showed significant differences (*p* < 0.0001)

As we observed a common overexpression of SEPTIN9 and CAPG at the protein level in dormant cells regardless of the tumour type, we investigated a potential functional impact by measuring the physical properties of the dormant cells compared to their parental counterpart cells in both MRD models. Indeed, these two proteins are involved in cytoskeleton organization [24–26], a feature involved in cell fate and adaptative mechanisms to the microenvironment [27, 28]. In addition, the cytoskeleton network may link mechanical properties with cell dormancy [27, 29]. Besides mechanical effects, multiple studies have described the role of the major cytoskeletal components such as actin filaments and microtubules, on the electrical properties of cells [30–32]. Interestingly, dynamic processes such as depolymerisation/polymerisation of microtubules and actin filaments have been described to impact the electrical signature of cancer cells [30, 31]. Thus, we performed impedance analyses to compare the electrical properties of cells from both MRD models. The required throughput for single-cell analysis was sustained using electrical impedance measurements in a microfluidic device. This micromachined impedance spectroscopy flow cytometer obtained different cell properties at dedicated measurement frequencies, e.g., size at frequencies between 0.1–1 MHz, membrane reactance at frequencies between 2–5 MHz, and cytoplasm conductance at frequencies higher than 20 MHz [33]. Therefore, we measured the response of each cell at 30 MHz to compare their cytoplasm conductance as a greater expression of SEPTIN 9 and CAPG, binding proteins involved in cytoskeleton remodeling, was detected in dormant cells from the 2 MRD models. To minimize possible errors due to device calibration, we normalized each measurement according to the median of the parental cells. DA1-3b/D365 leukemia (0.888 ± 0.007 normalized value ± standard error) and B16-F1GFP-D (0.84 ± 0.018 normalized value ± standard error) melanoma dormant cells exhibited lower normalized cytoplasm conductance than their parental counterpart cells, i.e., DA1-3b (1.000 ± 0.007) and B16-F1GFP-M (1.000 ± 0.020), repeated in 6 and 4 independent experiments for a total n = 14,379 and 3815 cells, respectively (Fig. 12). Both comparisons showed significant difference (p < 0.0001) between the parental and dormant cells in both MRD models (student t-test). Overall, these results reinforced the possible role of the cytoskeleton shape in mechanisms underlying the MRD process.

## Discussion

Investigation of the multiomics signatures of dormant melanoma and leukemia cells from the MRD syngeneic mouse models has provided crucial insights into the genetic and nongenetic mechanisms involved in tumour dormancy. Our multiomics approach revealed a specific genetic signature and highlighted its potential functional impact in both models. Although a discrete overlap among genes harbor mutations was observed between murine melanoma and leukemia dormancy models, a significant number of mutated genes appeared exclusively in dormant cells within both models. This singularity suggests potential resistance mechanisms against the microenvironment or therapies, indicating either the acquisition of advantageous mutations or preexisting clonal selection during the MRD process [10, 34–36]. The identification of exclusive mutated genes in parental cells reinforced the hypothesis linking the existence of original dormant clones to their parental counterparts [10, 35–37].

In the B16-F1 melanoma model, preexisting clone selection may be involved in MRD due to the lack of additional mutation acquisition among the 2nd generation postdormancy cells (B16-F1GFP-DB#1, B16-F1GFP-DB#2 and B16-F1GFP-DB#3). Our exome sequencing data of the DA1-3b leukemia cells preferentially support a mutation gain scenario. Even though our two MRD models were designed through a similar immunotherapy protocol, the MRD process may involve distinct mechanisms that lead to resistance. In human AML, the reappearance of leukemic disease can occur through mechanisms identical to those observed in the murine MRD model. Indeed, multiple studies through NGS deep sequencing revealed that an original clone or a subclone resistant to anticancer therapy, acquired new mutations, expanded and emerged as the predominant clone at relapse [35, 36]. Many lines of evidence have shown the existence of an ancestral, prediagnostic clone evolving and emerging as the major clone at relapse [35].

In human melanoma, the emergence of therapy-resistant tumour clones is frequently observed in melanoma patients and is responsible for tumour relapse and poor prognosis [10, 37]. We and others have previously shown that melanoma cells exhibit a high capacity for cellular plasticity, a nongenetic process that likely enables adaptation to the environment [38, 39]. In addition, cellular plasticity can control the phenotypic switch between stem-like and non-stem-like cancer cells [38]. As we have previously shown that dormant B16-F1GFP-D cells were enriched in stem-like phenotype cells, exome sequencing and associated VAF data reinforced the hypothesis that preexisting clones among parental cells may display plastic/stem cell features underlying the MRD process [12]. These observations call into question the mechanism underlying MRD in melanoma: is there a unique genetic or nongenetic mechanism or a combination of both as observed with our murine model?

As this study revealed specific gene signatures in melanoma dormant cells combined with previous data showing their stem-like phenotype, we hypothesized that both genetic and nongenetic mechanisms explain the MRD process. An elegant study recently highlighted a spatiotemporal map of the diversity and trajectories of melanoma cell states and proposed that the capacity for growth or metastasis is limited to distinct subsets of cells [40]. As these phenotypic behaviours can be dynamically acquired upon exposure to specific niche or microenvironment signals, these findings emphasize the reprogramming capacities of melanoma cells [40]. Although plasticity may be involved in the MRD mechanisms in melanoma, our results did not discriminate between genetic and nongenetic processes which could explain dormancy in the B16-F1 murine model. Indeed, the 2nd generation of B16-F1GFP-DB dormant cells did not feature any additional mutated genes without losing their stem-like cell properties. In addition, we previously showed that these brain site-derived melanoma cells modulate their transcriptome profile compared to the first generation of dormant cells without any additional genomic alterations [12, 38]. Thus, MRD could involve genetic and/or nongenetic mechanisms in melanoma.

Our multiomics analysis demonstrated that the genetic signature of dormant cells may have a functional impact. However, it is important to note that not all of the identified mutated genes from both dormancy models necessarily contribute to this functional impact. For a specific set of mutated genes in both MRD models, differential CNV alterations combined with epigenetic patterns and transcriptomic gene expression, highlighted possible functional impacts and involvement in MRD mechanisms. Although several mutated genes did not exhibit expression correlated with epigenetic repressive marks, a substantial number of genes harbouring mutations in dormant cells were expressed in agreement with the associated active histone code. In addition, the overexpression of mutated genes in dormant cells was mainly not associated with differential amplification or deletion between parental and dormant cells.

In regard to the murine leukemia model, combined epigenetic and transcriptomic analysis revealed that active histone marks were correlated with the overexpression of several mutated genes that are involved in metabolic pathways. Remarkably, the expression of these genes was not solely dictated by CNV alterations, underscoring a nuanced interplay between genetic and nongenetic factors in the context of MRD. Gene Ontology enrichment analysis revealed the potential functional roles of the expressed mutated genes, revealing a link to “cadherin binding” and “cell adhesion molecule binding,” whereas those in melanoma cells were associated with pathways involving “small molecules binding” and “nucleotide binding”.

To ascertain whether the “murine mutated gene signatures” revealed through our murine MRD models were relevant to the corresponding human disease, we analysed the genetic profiles of these genes in human AML and melanoma cohorts from public datasets. Although the dormant mutated gene signature revealed by the murine leukemia model did not precisely mirror, or did so only to a limited extent, the mutation patterns observed in human AML at diagnosis, revealed CNV alterations. This finding suggests a potentially significant role of these genes in human disease. In addition, because MRD or AML relapse datasets were not available, we could not verify the relevance of our signature in human samples during the progression of the disease. Nevertheless, *Idh1* was found to be mutated in murine leukemia dormant cells, and a mutated IDH1 gene is frequently observed in human AML at both diagnosis and MRD stages [15, 36].

In human melanoma samples, we confirmed the presence of similar mutated genes in primary and metastatic tumours. These observations reinforced the relevance of our findings for potential translational applications. While the frequency of “murine” dormant mutated genes was low in metastatic melanoma tumours without any expected “enrichment”, several genes exhibited CNV alterations with a significantly increased frequency compared to the primary stage. Although these observations reinforced the relevance of our findings for potential translational applications, the murine MRD models may also mimic disease features of one “single patient” regarding mutational patterns minimizing the utility of the identified mutated gene signatures related to dormancy.

In addition to the most commonly accepted explanation for the evolution of resistance involving genetic alterations [41], we focused our study on the nongenetic resistance of dormant cells. Indeed, recent and robust studies indicate that drug-tolerant persister phenotype(s) can be transiently acquired through nonmutational mechanisms [10, 42–44]. The proteomic analysis of dormant cells revealed that dysregulated protein expression was primarily driven by nongenetic mechanisms in both murine MRD models. In leukemia dormant cells, enrichment analysis identified pathways associated with cellular metabolism, providing valuable insights into the molecular mechanisms governing dormant cells. Similarly, dysregulation of proteins in dormant melanoma cells was influenced mainly by nongenetic factors, and enrichment pathway analysis revealed its involvement in cellular metabolism. However, dormant melanoma cells also exhibit a lack of differentiation and dysregulated cytoskeleton organization. This study enables a comparison between the dormant cell signature from our MRD models and those designed in previous studies involving chemotherapy and human cells from patients [10, 45].

Interestingly, several identified genes and/or pathways, were common to those identified in signatures from several MRD melanoma models such as the neural crest stem cells, MITF targets, differentiation/pigmentation and invasion [10]. In the same way, in regard to the PDX (patient-derived xenograft) AML MRD models from other studies, persistent cells following chemotherapy, exhibited metabolic reprogramming as noted in our dormant leukemia model [8]. In addition, the GO enrichment pathway analysis revealed a significant involvement of dysregulated proteins observed in dormant DA1-3b/D365 cells, in several cellular biosynthesis processes. Interestingly, an elegant study revealed a “dormant cell signature” in hematopoietic stem cells (HSC) with implications for biosynthesis pathways during the transition from dormant to active HSCs [46]. Similarly, a recent study, revealed that these “dormant signature” could (i) discriminate functionally distinct cell compartments in the leukemic stem cell pool from those in AML patients and (ii) provide crucial insights into the cell fate trajectories of these potentially resistant cells [47].

Overall, these observations reinforce our findings and highlight the common features underlying the MRD process regardless of the nature of anticancer treatment that leads to dormancy. Indeed, our syngeneic MRD models were designed through immunotherapy while MRD models in other studies were obtained under chemotherapy [8, 10–12]. A very limited number of studies have described MRD mouse models and mainly used PDX models devoid of immune system implications. This study conducted in syngeneic models of leukemia and melanoma may decipher potential immune-related mechanisms of dormancy. As previously shown in our leukemia MRD model, overexpression of the immune inhibitory ligands PD-L1 enhanced resistance to CD8 T lymphocytes [11]. Conversely, the mechanisms elucidated in our syngeneic MRD models, that are distinct from those highlighted in other MRD models, may be implicated within the context of immune system resistance.

Interestingly, this study revealed the common differential expression of 11 proteins in leukemia and melanoma dormancy models. This suggests a potential “general” signature for MRD. SEPTIN-9 and CAPG consistently exhibited increased expression in both pathologies, indicating a shared structural organization in the cytoskeleton during tumour dormancy. In addition, several dysregulated proteins in dormant cells were significantly enriched in cytoskeleton-related pathway. Remarkably, many lines of evidence have revealed that the mechanical properties dictated by the cytoskeleton may impact cell fate including tumor dormancy or phenotypes better suited to a novel environment [27–29]. Our physical properties investigation revealed a common “electrical” signature in dormant cells from the two MRD models. The cytoplasm conductance of the dormant cells was significantly lower, signifying a profound change in the structural organization of microtubules and actin filaments. Although depolymerized/polymerized actin filaments or microtubules have been described to have contradictory impacts on conductance or electrical properties [30, 31], this study highlights a significant role of the cytoskeleton underlying MRD processes across various cancer types.

The use of a syngeneic model provides a significant advantage in cancer research. This approach minimizes interindividual genetic variabilities, thereby enhancing the reliability of outcomes and the validity of deductions drawn from the study. By exploring the genetic and proteomic signatures of dormant cells in the context of immunotherapy, our study offers new and valuable insights. Conclusions regarding the differences or similarities in signatures can inform therapeutic strategies for MRD treatment across a spectrum of cancer types.

Moreover, the rich multiomics approach enables a comprehensive exploration of the molecular mechanisms governing MRD. Through the combination of genomic, transcriptomic, proteomic, and epigenomic datasets, this methodology affords a holistic and exhaustive view of the biological landscape of MRD, offering valuable insights for the development of targeted strategies [48]. Despite these strengths, our study lacked single-cell analysis, which could constrain the resolution and comprehension of phenotypic variations within cellular populations [36]. Incorporating this approach would have enabled a more precise characterization of the specific cell subpopulations implicated in MRD [49]. In addition, although the cellular models employed in the study consisted of cells amplified in vitro immediately after the in vivo dormancy state and were thus, in a proliferative state, they still manifested features of tumour dormancy that render them relevant for MRD investigation [11, 12]. Our murine models showed a decrease in the proliferation of dormant cells compared to the parental ones, alongside a noted lack of differentiation, resistance to the immune system and/or a stem cell-like phenotype according to the pathology involved [11–13]. While these cells may not be in a quiescent state, they do exhibit a dormant state that may reflect a late stage of MRD or even early events of relapse. In addition, several MRD models highlight the nonquiescence state of residual cells and indicate “functional dormancy” which may illustrate the immune system interactions during MRD [7, 8, 10, 50]. Thus, our models may also reflect MRD mechanisms in an “active” state of dormant cells (as shown by the enrichment of biosynthetic processes) or a state of postimmune system pressure.

## Conclusions

In summary, despite its limitations, this study offers a comprehensive and innovative perspective on MRD research, leveraging syngeneic models and considering the immune context. These results provide a robust groundwork for future investigations and hold substantial potential for the development of more effective strategies against MRD in cancer. Our findings suggest that murine models closely mimic patient conditions, providing valuable insights into the genetic and protein signatures of MRD. Understanding these mechanisms could guide the development of therapeutic strategies to address MRD in leukemia and melanoma patients.

## Materials and methods

### MRD leukemia DA1-3b and B16-F1 melanoma mouse models

Murine leukemic myeloid cells (DA1-3b parental cells and DA1-3b/D365 dormant cells) and melanoma cells (B16-F1GFP-M parental cells, B16-F1GFP-D, B16-F1GFP-DB#1, B16-F1GFP-DB#2, and B16-F1GFP-DB#3 dormant cells) were isolated as previously described [11, 12]. Briefly, dormant cells were isolated from MRD syngeneic mouse models based on immunotherapy, capturing distinct dormancy stages (Day 60 and Day 365 for the leukemia model, and Day 365 and “2nd generation in brain site” for the melanoma model). The cells were amplified in vitro and stored in liquid nitrogen until subsequent analysis.

### Array-comparative genomic hybridization analysis

DNA extraction from frozen samples was carried out following the manufacturer’s recommendations QIAmp DNA Mini Kit, Qiagen Valencia, CA, USA). The quantity and quality of the extracted DNA were were assessed on a NanoDrop platform (Thermo Fisher Scientific, Waltham, MA, USA) and by gel electrophoresis. CGH array analysis was achieved using pangenomic arrays consisting of 60-mer oligonucleotides (027411_D_F_20150623 design version, Mouse Genome 180 K CGH array, Agilent Technologies Santa Clara, CA, USA). The arrays were scanned using an Agilent G2505B scanner, and data were analysed with Agilent Feature Extraction Software (v10.7.3.1) against the mm9 (mm9: NCBI37) mouse genome assembly. The array data have been deposited in the Gene Expression Omnibus (GEO) under accession number GSE250172 for public accessibility. CNVs were detected using the Aberration Detection Method 2 (ADM2) algorithm. CNVs was called either gain or deletion according to the log2 ratio distribution analysed with Genomic Workbench software (v5.0.14).

### Whole exome sequencing analysis

DNA was extracted as described in section “array-comparative genomic hybridization analysis”. Library preparation was performed using the SureSelect Target Enrichment System (Agilent Technologies, USA), with 1 µg of genomic DNA fragmented and the library purified and size-selected using AMPure XP beads (Beckman Coulter Life Sciences, USA). Following ligation of Ion Xpress barcodes and P1 adapters, the libraries were amplified.. The amplified DNA fragments underwent hybridization to biotinylated RNA library baits and subsequent capture using streptavidin-coated magnetic beads. Quality assessment of the captured library fragments was performed on a 2100 Bioanalyzer (Agilent Technologies, USA). Template preparation utilized Ion PI™ Hi-Q™ chemistry (Life Technologies, USA), with 50 pM of each library loaded onto an Ion Chef™ Instrument (Life Technologies, USA) for template enrichment. Templating efficiency of the Ion spheres was evaluated using a Qubit™ 2.0 fluorometer (Thermo Fischer Scientific, USA). Prepared libraries were loaded onto Proton PI chips v3 (two samples/chip) and sequenced on an Ion Proton using PI™ Hi-Q™ sequencing 200 chemistry (Life Technologies, USA) with a read length of 260 bp and 520 flow cycles. Data analysis involved the Ion Torrent platform-specific pipeline software (Torrent Suite v4.0) for read separation, sequence alignment to the mm10 mouse genome reference, target-region coverage analysis, and removal of low-quality reads. Exome sequencing data are publicly accessible through Sequence Read Archive (SRA) under the PRJNA1103364 bioproject number. The alignment file from the Torrent Suite was transferred to Ion Reporter (Ion Reporter v4.0) for variant file generation using default parameters. A total of 218, 914 variants (insertions/deletions/SNPs) were detected in the total samples.

### Targeted sequencing analysis

DNA was extracted as described in section “array-comparative genomic hybridization analysis”. AmpliSeq libraries were prepared using the Ion AmpliSeq Library Kit 2.0 and Ion AmpliSeq Custom Panel (Life Technologies). AmpliSeq technologies were used to design a custom NGS library including 190 amplicons in two pools, covering all the targets of interest (30.6 kb covered at 100%). The targets of interest were selected from the whole exome sequencing results, and filtered by the effects of the variants on genes, transcripts, protein sequences and regulatory regions. These effects were calculated using the Ensembl VEP and SIFT tools (https://www.ensembl.org/info/docs/tools/vep/index.html). Ten nanograms of each DNA sample served as the template for library preparation. Quality control of all libraries was conducted using the Agilent Bioanalyzer with high sensitivity chips. Template dilutions were calculated post-normalization of library concentrations to 100 pM using the Ion Library Equalizer Kit (Life Technologies). Library templates underwent clonal amplification using the Ion One Touch 2TM system as per the manufacturer’s instructions. Following enrichment of recovered template-positive Ion Sphere Particles, samples were sequenced using Ion 318 v2 chips on the Ion PGM System or Ion 530 chips on the Ion S5XL system (Thermo Fisher Scientific). Data analysis utilized the Torrent Suite Software v.5.2.2 (Thermo Fisher Scientific) with alignment to the mm10 mouse genome. Variant calling was optimized, achieving a mean depth of 5000 reads for each sample.

### ChIP sample preparation for sequencing

Cells were treated with 1% formaldehyde for 10 min at room temperature to cross-link proteins and DNA. The reaction was quenched with 125 mM glycine for 5 min, followed by cell collection and lysis in the following buffer [HEPES/KOH 0.01 M pH 7.9, KCl 0.01 M, MgCl_2_ 0.0015 M and 1 × protease inhibitor cocktail (Sigma-Aldrich)]. After centrifugation at 10,000×*g* for 10 min at 4 °C, the supernatant containing chromatin was collected. Chromatin was sheared by sonication at 4 °C using a Bioruptor 300 to generate fragments of approximately 200–400 bp in length, with 15 cycles of 30 s ON/OFF at the highest setting. For immunoprecipitation, 100 µL of the chromatin supernatant was incubated overnight at 4 °C on a rotating wheel with specific antibodies against H3K9 (Diagenode), H3K27 (#, Active Motif), or H3K4 (Diagenode) trimethylation, using 0.25 μg of antibody for 0.1 A_260nm_. Control experiments included the use of an equivalent amount of irrelevant control IgGs (Millipore). An aliquot of the same amount was saved as the input sample and stored overnight at − 20 °C. The next day, immune complexes were incubated with 100 µL of magnetic beads for 3 h at 4 °C under rotation. Beads were washed sequentially with low salt wash buffer, high salt wash buffer, and TE buffer 1X pH 8.0. Following each wash, beads were centrifuged at 960×*g* for 3 min at room temperature. After removing the supernatant, immune complexes were eluted with 210 µL of elution buffer and incubated for 15 min at 65 °C with stirring. Eluted material was collected by centrifugation at 16,000×*g* for 1 min at room temperature. The immunoprecipitated material was eluted at room temperature in elution buffer (100 mM NaHCO_3_, 1% SDS), and the crosslinking reactions were reversed by adding 100 mM NaCl and incubating at 65 °C overnight. The eluted material was then treated with Q-Protease (Qiagen) and RNase H to remove protein and RNA, respectively, and the enriched genomic DNA fragments were purified according to the Macherey Nagel protocol (Kit Nucleospin Gel and PCR Clean up), eluted in 35 µL of sterile water and stored until sequencing.

### Chip-Seq analysis

The DNA samples were sequenced at GATC (Eurofins Genomics, Ebersberg, Germany). Illumina sequencing libraries were prepared according to ISO 17025 standards and applied to an Illumina HiSeq 2500 platform (Illumina, San Diego CA, USA) for 50 bp paired-end sequencing, ensuring a minimum of 30 Mb per sample. The ChIP-Seq data (fastq) are publicly accessible through the Sequence Read Archive (SRA) under the PRJNA1054015 bioproject number. Analysis of the aligned data was conducted using the Partek Genomics Suite ChIP-Seq workflow (version PGS7.20.0831 for Windows). Standard methods were employed for data importation and quality control assessment. Peak detection allowed the identification of enriched regions, encompassing both novel and known genomic loci, thereby pinpointing potential targets.

### Array gene expression analysis

Total RNA extraction was conducted using the RNeasy Mini Kit (QIAGEN, Courtaboeuf, France) as per the manufacturer’s protocol, including additional DNase treatment. The yield and quality of total RNA were evaluated using an Agilent 2100 Bioanalyzer (Agilent Technologies, Massy, France). Gene expression analysis was performed using one-color whole Mouse 8 × 60 k microarrays (074809_D_F_20150624 slides, Agilent Technologies). cRNA labelling, hybridization, and detection followed standard protocols provided by Agilent Technologies. Cyanine 3-labelled cRNA was synthesized from 50 ng of total RNA using a low-input QuickAmp labelling kit, with RNA Spike-In serving as a positive control for labelling and amplification steps. Purified labelled cRNAs (600 ng each) were hybridized and washed according to manufacturer’s instructions, followed by scanning on an Agilent G2505C scanner. Data extraction was performed using Agilent Feature Extraction Software© (FE version 10.7.3.1). The microarray data are publicly accessible through the Gene Expression Omnibus (GEO) series accession number GSE250145. Statistical analysis and filtering were conducted using Genespring® software version GX13.0 (Agilent Technologies).

### Sample preparation for MS analysis

HPLC grade acetonitrile (ACN), water, and analytical reagent (AR)-grade trifluoroacetic acid (TFA) were from Biosolve B.V (Valkensvaard, Netherlands). Ammonium bicarbonate (NH4HCO3), DL-dithiothreitol (DTT), iodoacetamide (IAA), urea, tris(hydrochloric acid) (Tris–HCl), and sodium chloride (NaCl) were obtained from Sigma-Aldrich (Saint-Quentin Fallavier, France). Sodium dodecyl sulfate (SDS) was purchased from Bio-Rad (Marnes La Cocquette, France), and AR-grade formic acid (FA) wasfrom Fluka. Thiourea and urea were obtained from Fluka and Euromedex, respectively. Sequencing grade modified porcine trypsin was obtained from Promega (Trypsin Gold, Mass Spectrometry Grade, Charbonnières-les-Bains, France). Protein extraction and digestion were carried out following established protocols [51]. Cells were lysed in a buffer comprising 4% SDS and 100 mM DTT in 100 mM Tris–HCl (pH 7.6), followed by sonication for three 30-s cycles at 500 W and 20 kHz. Centrifugation at 14,000×*g* for 10 min at room temperature was then performed to pellet cell debris, and the supernatant containing proteins was collected. The Filter-Aided Sample Preparation (FASP) method was employed using Amicon Ultra-0.5 mL 10 kDa filters, following the protocol detailed in [51]. Protein digestion was conducted with trypsin (Promega, Gold MS, mass spectrometry grade) at a concentration of 20 µg/mL in 50 mM NH4HCO3, with an overnight incubation at 37 °C. Peptide digests were subsequently collected via centrifugation, and the filters were rinsed with 50 µL of 0.5 M NaCl. After adding 5% TFA, the digests were desalted using Millipore ZipTip C18 devices. The desalted solution was dried and reconstituted in water with 0.1% formic acid and 2% acetonitrile, ready for LC‒MS/MS analysis.

### LC–MS/MS analysis

Samples were analysed by online reversed-phase chromatography using a Thermo Scientific Proxeon EASYnLC 1000 system. The system was equipped with a precolumn (Acclaim Pepmap, 75 µm ID × 2 cm, Thermo Scientific, Waltham, MA, USA) and a C18 packed-tip column (Acclaim PepMap, 75 µm ID × 50 cm, Thermo Scientific, Waltham, MA, USA). Peptide separation was achieved with a gradient of acetonitrile (ACN) ranging from 5 to 35% over 120 min at a flow rate of 300 nL/min. The LC eluent was electrosprayed directly from the analytical column, with a voltage of 1.7–2.6 kV applied to the nanospray source. The chromatography system was connected to a Thermo Scientific Q Exactive mass spectrometer, set to operate in data-dependent acquisition mode, to target the Top 10 most intense ions. Survey scans were conducted at a resolving power of 70,000 FWHM (m/z 400) in positive mode, with an automatic gain control (AGC) target of 3e6. The default charge state was set to two, with unassigned and singly charged states being excluded, and dynamic exclusion enabled for 20 s. The scan range for survey scans was 300–1600 m/z. For data-dependent MS/MS (ddMS2) analysis, the scan range was set from 200 to 2000 m/z, with one microscan acquired at 17,500 full width at half maximum (FWHM). An isolation window of 4.0 m/z was used for selecting precursor ions.

### Data analysis MS

All MS data were processed using MaxQuant software (version 1.5.6.5) with the Andromeda search engine. Proteins were identified by searching MS and MS/MS data against the Mus musculus database (50,306 sequences). Trypsin specificity was selected for the digestion mode, with N-terminal acetylation and methionine oxidation as variable modifications. Carbamidomethylation of cysteines was set as a fixed modification, allowing up to two missed cleavages. An initial mass accuracy of 6 ppm was chosen for MS spectra, with a minimum of two peptides and at least one unique peptide per protein. The MS/MS tolerance was set to 20 ppm for HCD data. The false discovery rate (FDR) for peptide spectrum matches (PSMs) and protein identification was set to 0.01. Label-free quantification (LFQ) of proteins was conducted using the MaxLFQ algorithm integrated into MaxQuant with default parameters. Identified proteins were further analysed using Perseus software (version 15.6.0). The data file containing identification information was filtered to remove hits to the reverse database, proteins identified only with modified peptides, and potential contaminants. The LFQ intensity values were logarithmically transformed (log2[x]). Categorical annotation of rows was used to define different groups of replicates. For statistical analysis, only significant proteins according to the Student’s t-test were considered.

### GO pathway enrichment analysis

The Protein–Protein Interaction (PPI) network and Gene Ontology (GO) analyses of differentially expressed proteins and proteins associated with mutated genes were constructed using STRING version 10.0 (http://string-db.org/). Pathway enrichment analyses were visualized as bubble plots generated with GraphPad Prism version 10.2.0.

### Physical properties measurements

Electrical impedance measurements were conducted using a hybrid micro electro-mechanical systems (MEMS)/microfluidic device fabricated on a silicon-on-insulator wafer with a two-mask process [52]. This device featured an embedded microfluidic channel with 3D facing electrodes on each side for electrical measurements. The electrical properties of the cells influenced the current passing between the electrodes as they flowed through the channel [53]. The measurements were carried out with a lock-in amplifier (HF2LI, Zurich Instruments) and a trans-impedance amplifier (1 k gain, HF2TA, Zurich Instruments) using a 1-V_rms_ driving signal. The flow rate was set at 3 µL/min, controlled by a pressure pump (LineUpTM Push–Pull, Fluigent) connected to the outlet. Changes in the real and imaginary components of the current, amplified to a potential difference, were recorded as each cell passed between the electrodes. Data processing was performed using a custom Python script to extract the response of each cell. To account for potential variations in device characteristics, the cell responses were normalized against the median response of the parental cells. Statistical analyses were performed using Student's t-tests.

### Analysis of public datasets

DNA-seq, RNA-seq, and CNV datasets from human AML samples and melanoma [16, 22, 23, 54] are available in the public domains cBioPortal [55–57] and NIH National Cancer Institute GDC Data portal. Version 1.0.

### Supplementary Information

Additional file 1: Table S1. The following raw data were obtained: exome sequencing data, histone epigenetic marker data, transcriptomic gene expression data and proteomic MS data. a) Description of mutation types in the indicated genes in DA1-3b and B16-F1 cells. b) Multiomics data analysis from CNV, histones marks, and transcriptomic gene expression data for the indicated mutated genes in both MRD models. c) Percentages of the indicated mutated genes and the associated CNVs in the human AML and melanoma cohorts. d) Percent of the 50 most frequently mutated genes and the associated CNVs in the human AML and melanoma cohorts. The values are represented by a distinct colour gradient. For human melanoma samples, the results are represented according to the stage of disease progression, i.e., primary or metastatic tumours. The data were extracted from the public domain GDC portalversion 1.0. e) Proteomic MS analysis and resulting LFQ data in both MRD models. f) Multiomics data analysis of CNV, histones marks, and transcriptomic gene expression data for the indicated dysregulated proteins in the leukemia model. g) Multiomics data analysis of CNV, histones marks, and transcriptomic gene expression data for the indicated dysregulated proteins in a melanoma model. h) Multiomics data analysis of CNV, histones marks, and transcriptomic gene expression data for the 11 common dysregulated proteins in both MRD models

Additional file 2: Figure S1. Karyotype profiles of parental and dormant cells from the 2 MRD models. In the following order, the conditions were B16-F1GFP-M, B16-F1GFP-D, B16-F1GFP-DB#1, B16-F1GFP-DB#2, B16-F1GFP-DB#3, DA1-3b, DA1-3b/D60, and DA1-3b/D365 cells

Additional file 3: Figure S2. Multiomics data analysis of CNV, histones marks, and transcriptomic gene expression data for the indicated exclusively expressed proteins in dormant or parental cells from the MRD melanomaand leukemiamodels

## Data Availability

The datasets generated and/or analysed during the current study are available as indicated in “Materials and methods” section. The remaining data generated or analysed during this study are included in this published article [and its supplementary information files].

## Abbreviations

ACN: Acetonitrile
AGC: Automatic gain control
AML: Acute myeloid leukemia
CGH: Comparative genomic hybridization
CHiP-seq: Chromatin immunoprecipitation followed by sequencing
CNV: Copy number variation
DNA: Deoxyribonucleic acid
DTT: Dithiothreitol
FA: Formic acid
FWHM: Full width at half-maximum
GM-CSF: Granulocyte–macrophage colony-stimulating factor
GO: Gene ontology
GEO: Gene expression omnibus
IAA: Iodoacetamide
IL-12: Interleukin-12
LFQ: Label-free quantification
MEMS: Micro electro-mechanical systems
MRD: Minimal residual disease
MS: Mass spectrometry
PPI: Protein–protein interaction
RNA: Ribonucleic acid
SDS: Sodium dodecyl sulfate
SIFT: Sorting Intolerant From Tolerant
SMG: Significantly mutated gene
SNP: Single nucleotide polymorphism
SNV: Single-nucleotide variant
VAF: Variant allele frequency
WES: Whole exome sequencing
