# Supplementary material for: Deciphering genetic and nongenetic factors underlying tumour dormancy: insights from multiomics analysis of two syngeneic MRD models of melanoma and leukemia
Source: Biol Res. 2024 Sep 3;57:59. doi: 10.1186/s40659-024-00540-y (PMC11370043; doi:10.1186/s40659-024-00540-y)
Supplement: Supplementary file 3 — Additional file 3: Figure S2. Multiomics data analysis of CNV, histones marks, and transcriptomic gene expression data for the indicated exclusively expressed proteins in dormant or parental cells from the MRD melanomaand leukemiamodels [file 40659_2024_540_MOESM3_ESM.zip › Figure S2B R1.pdf]

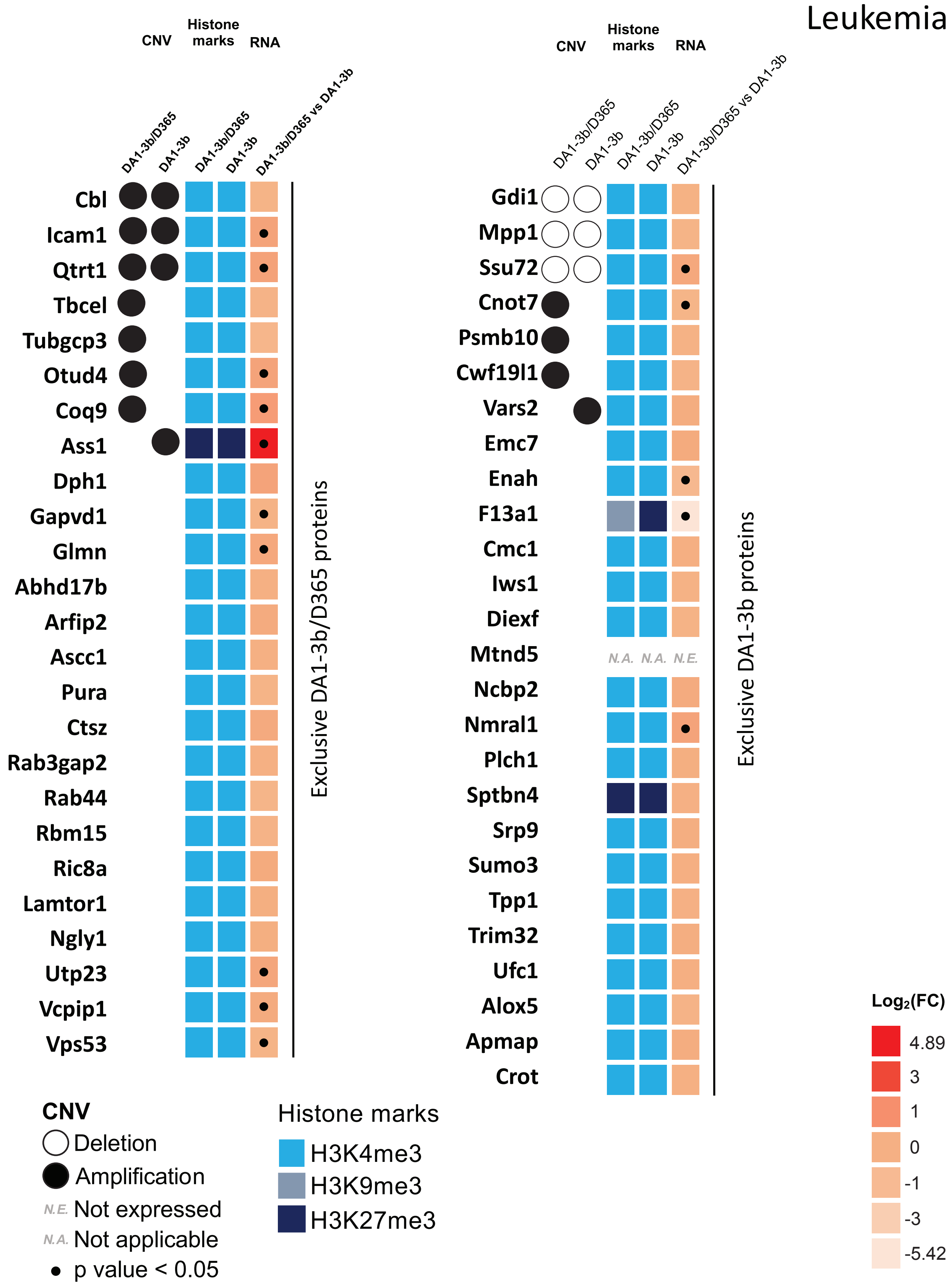

Leukemia
CNV
Histone marks
RNA
DA1-3b/D365
DA1-3b
DA1-3b/D365
DA1-3b
DA1-3b/D365 vs DA1-3b
Cbl
Icam1
Qtrt1
Tbcel
Tubgcp3
Otud4
Coq9
Ass1
Dph1
Gapvd1
Glmn
Abhd17b
Arfip2
Ascc1
Pura
Ctsz
Rab3gap2
Rab44
Rbm15
Ric8a
Lamtor1
Ngly1
Utp23
Vcpip1
Vps53
Exclusive DA1-3b/D365 proteins
Gdi1
Mpp1
Ssu72
Cnot7
Psmb10
Cwf19l1
Vars2
Emc7
Enah
F13a1
Cmc1
Iws1
Diexf
Mtnd5
N.A.
N.A.
N.E.
Ncbp2
Nmral1
Plch1
Sptbn4
Srp9
Sumo3
Tpp1
Trim32
Ufc1
Alox5
Apmap
Crot
Exclusive DA1-3b proteins
CNV
Deletion
Amplification
N.E. Not expressed
N.A. Not applicable
p value < 0.05
Histone marks
H3K4me3
H3K9me3
H3K27me3
Log2(FC)
4.89
3
1
0
-1
-3
-5.42
