# Supplementary figures and images for: Deciphering genetic and nongenetic factors underlying tumour dormancy: insights from multiomics analysis of two syngeneic MRD models of melanoma and leukemia
Source: Biol Res. 2024 Sep 3;57:59. doi: 10.1186/s40659-024-00540-y (PMC11370043; doi:10.1186/s40659-024-00540-y)

# Melanoma

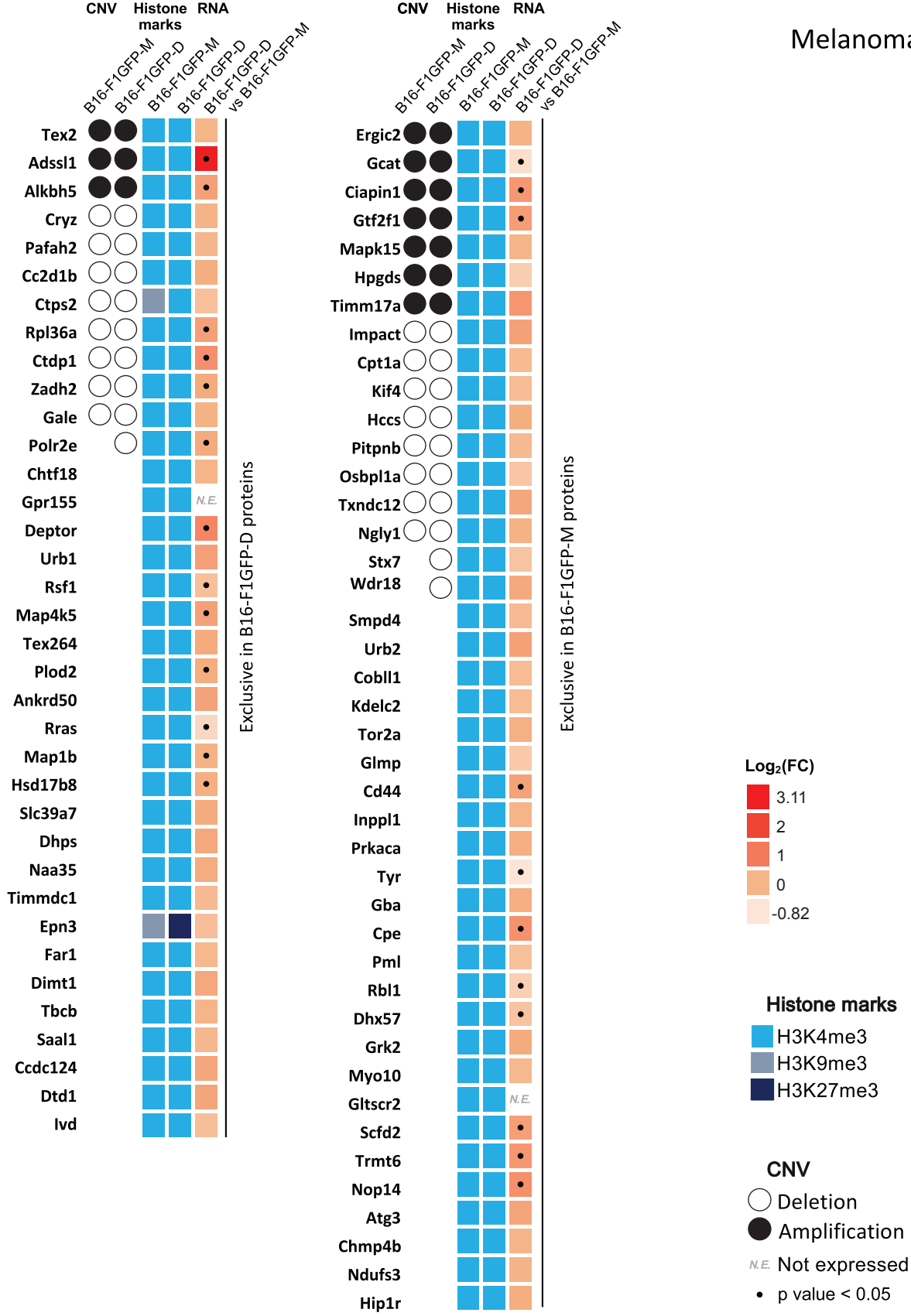

Supplement: Supplementary file 3 — Additional file 3: Figure S2. Multiomics data analysis of CNV, histones marks, and transcriptomic gene expression data for the indicated exclusively expressed proteins in dormant or parental cells from the MRD melanomaand leukemiamodels [file 40659_2024_540_MOESM3_ESM.zip › Figure S2A R1.pdf]
