# Supplementary material for: Deciphering genetic and nongenetic factors underlying tumour dormancy: insights from multiomics analysis of two syngeneic MRD models of melanoma and leukemia
Source: Biol Res. 2024 Sep 3;57:59. doi: 10.1186/s40659-024-00540-y (PMC11370043; doi:10.1186/s40659-024-00540-y)
Supplement: Supplementary file 2 — Additional file 2: Figure S1. Karyotype profiles of parental and dormant cells from the 2 MRD models. In the following order, the conditions were B16-F1GFP-M, B16-F1GFP-D, B16-F1GFP-DB#1, B16-F1GFP-DB#2, B16-F1GFP-DB#3, DA1-3b, DA1-3b/D60, and DA1-3b/D365 cells [file 40659_2024_540_MOESM2_ESM.pdf]

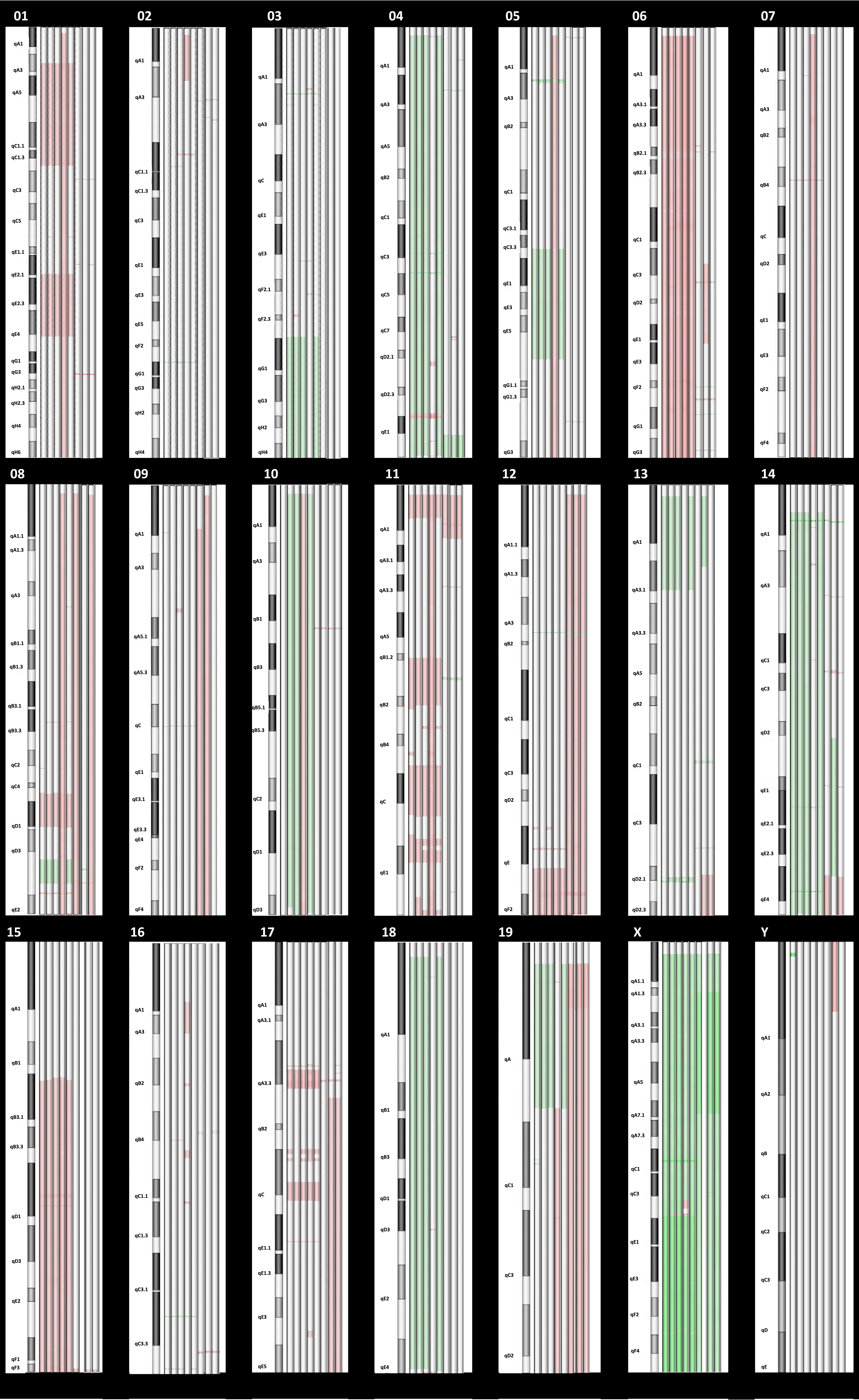
01
qA1
qA3
qA5
qC1.1
qC1.3
qC3
qC5
qE1.1
qE2.1
qE2.3
qE4
qG1
qG3
qH2.1
qH2.3
qH4
qH6
02
qA1
qA3
qC1.1
qC1.3
qC3
qE1
qE3
qE5
qF2
qG1
qG3
qH2
qH4
03
qA1
qA3
qC
qE1
qE3
qF2.1
qF2.3
qG1
qG3
qH2
qH4
04
qA1
qA3
qA5
qB2
qC1
qC3
qC5
qC7
qD2.1
qD2.3
qE1
05
qA1
qA3
qB2
qC1
qC3.1
qC3.3
qE1
qE3
qE5
qG1.1
qG1.3
qG3
06
qA1
qA3.1
qA3.3
qB2.1
qB2.3
qC1
qC3
qD2
qE1
qE3
qF2
qG1
qG3
07
qA1
qA3
qB2
qB4
qC
qD2
qE1
qE3
qF2
qF4
08
qA1.1
qA1.3
qA3
qB1.1
qB1.3
qB3.1
qB3.3
qC2
qC4
qD1
qD3
qE2
09
qA1
qA3
qA5.1
qA5.3
qC
qE1
qE3.1
qE3.3
qE4
qF2
qF4
10
qA1
qA3
qB1
qB3
qB5.1
qB5.3
qC2
qD1
qD3
11
qA1
qA3.1
qA3.3
qA5
qB1.2
qB2
qB4
qC
qE1
12
qA1.1
qA1.3
qA3
qB2
qC1
qC3
qD2
qE
qF2
13
qA1
qA3.1
qA3.3
qA5
qB2
qC1
qC3
qD2.1
qD2.3
14
qA1
qA3
qC1
qC3
qD2
qE1
qE2.1
qE2.3
qE4
15
qA1
qB1
qB3.1
qB3.3
qD1
qD3
qE2
qF1
qF3
16
qA1
qA3
qB2
qB4
qC1.1
qC1.3
qC3.1
qC3.3
17
qA1
qA3.1
qA3.3
qB2
qC
qE1.1
qE1.3
qE3
qE5
18
qA1
qB1
qB3
qD1
qD3
qE2
qE4
19
qA
qC1
qC3
qD2
X
qA1.1
qA1.3
qA3.1
qA3.3
qA5
qA7.1
qA7.3
qC1
qC3
qE1
qE3
qF2
qF4
Y
qA1
qA2
qB
qC1
qC2
qC3
qD
qE
